# Mitochondrial DNA duplication, recombination, and introgression during interspecific hybridization

**DOI:** 10.1038/s41598-021-92125-y

**Published:** 2021-06-16

**Authors:** Silvia Bágeľová Poláková, Žaneta Lichtner, Tomáš Szemes, Martina Smolejová, Pavol Sulo

**Affiliations:** 1 Department of Biochemistry, Faculty of Natural Sciences, Comenius University, Ilkovičova 6, Bratislava, 842 15 Slovakia; 2 Comenius University Science Park, Bratislava, 841 04 Slovakia; 3 Department of Molecular Biology, Faculty of Natural Sciences, Comenius University, Bratislava, 842 15 Slovakia; 4 Geneton s.r.o., Galvaniho 7, Bratislava, 821 04 Slovakia; 5 Present Address: Department of Membrane Biochemistry, Centre of Biosciences, Slovak Academy of Sciences, Bratislava, 84005 Slovakia

**Keywords:** Evolution, Genetics

## Abstract

mtDNA recombination events in yeasts are known, but altered mitochondrial genomes were not completed. Therefore, we analyzed recombined mtDNAs in six *Saccharomyces cerevisiae* × *Saccharomyces paradoxus* hybrids in detail. Assembled molecules contain mostly segments with variable length introgressed to other mtDNA. All recombination sites are in the vicinity of the mobile elements, introns in *cox1*, *cob* genes and free standing *ORF1*, *ORF4.* The transplaced regions involve co-converted proximal exon regions. Thus, these selfish elements are beneficial to the host if the mother molecule is challenged with another molecule for transmission to the progeny. They trigger mtDNA recombination ensuring the transfer of adjacent regions, into the progeny of recombinant molecules. The recombination of the large segments may result in mitotically stable duplication of several genes.

## Introduction

Hybrids between *Saccharomyces* species occur frequently in nature as a number of hybrids have been reported among wine and beer strains^1–6^. Most lager beer strains are hybrids between *S. cerevisiae* and *S. eubayanus*, combining the ability to produce ethanol with cryotolerance^6–8^. Some *S. cerevisiae* × *S. kudriavzevii* strains are associated with beer, but most of them are associated with wine, where they provide unique flavor^8,9^. *S. eubayanus* and *S. uvarum* hybrids have been associated with sparkling wine, cider fermentation, and, in some cases, with the production of off-flavors in breweries^8,10,11^. Interspecific hybrids among *Saccharomyces* species can also be readily obtained in the laboratory^9,12–14^. Hybrid species initially contain one nuclear genome from each parent; although, in older hybrids, the two parental genomes often undergo nonreciprocal exchanges accompanied by loss of genes, chromosomal segments, or even complete chromosomes, producing various chimaeras from which novel lineages might emerge^9,14–16^.

In contrast to the heterozygosity in the nuclear genomes, the mitochondrial genomes were reported in early works to be inherited only from one parent; although, in most cases the inheritance of mitochondrial DNA (mtDNA) was inferred from restriction patterns or from the sequence of a single mitochondrial gene^3,13,17–21^.

However, it has been known for many years that when two yeast cells containing mtDNAs are mated, the mtDNAs are biparentally inherited and undergo recombination followed by rapid fixation of a single mitochondrial haplotype^15,22,23^. Therefore, it was surprising that recombinant mtDNA molecules were not observed in early interspecific hybrids.

In *S. cerevisiae*, after mating, two haploid mtDNAs repeatedly recombine, and the cell reaches the homoplasmy usually in less than 20 generations. This is apparently related to the bottleneck effect and transmission of only a few concatemers to the nascent bud^24,25^. Recombination between parental mtDNAs can occur during the heteroplasmic transition, and a complete mitochondrial recombination map has been released from genome-wide sequencing. Recombination hotspots were preferentially localized in intergenic and intronic regions^26^. mtDNA recombination was also inferred from restriction fragment length polymorphism (RFLP) in *S. cerevisiae* intraspecific crosses^27^.

Later on, elevated nucleotide diversity was found in recombination hotspots of some genes, most of all *cox2*-*ORF1* in other *Saccharomyces* and also in hybrid species^28–30^. Recently, mtDNA recombination events were also reported in a study generating hybrid *Saccharomyces* allopolyploids of at least six species. Genome-wide sequencing revealed that during interspecific hybridization hybrids inherited mostly one of the two parental mitotypes that was quickly fixed, except for *S. kudriavzevii* × *S. mikatae* and *S. cerevisiae* × *S. uvarum* allotetraploids and the six species hybrids, which were all heteroplasmic. In addition, in the *S. kudriavzevii* × *S. mikatae* hybrid, the portion of the *cox1* gene from *S. mikatae* seemed to have been introduced into the *S. kudriavzevii* mtDNA. Unfortunately, these recombined mitochondrial genomes were not completed^9^.

In summary, several mtDNA recombination events were reported, but altered mitochondrial genomes were not assembled. Therefore, the aim of this work was to analyze mtDNA transmission and the rate of recombination in *S. cerevisiae* × *S. paradoxus* hybrids in detail.

## Results

### Interspecific hybrids

To avoid any alteration due to auxotrophic mutations and to mimic the condition in nature (prototrophy), our first intention was to mate type strains of *S. cerevisiae* CBS 1171 to *S. paradoxus* CBS 432^31^ as well as to *S. paradoxus* CBS 2908. However, CBS 1171 is an extremely poor sporulant; therefore, we used *S. cerevisiae* CCY 21-4-96 instead. *S. cerevisiae* and *S. paradoxus* differ in two basic phenotypic traits. The *S. cerevisiae* CCY 21-4-96 strain grows at 37 °C and produces rough colonies, whereas *S. paradoxus* strains do not grow at 37 °C and produce smooth colonies. Upon spore-to-spore mating, smooth colonies were prepared capable to grow at 37 °C from two independent crosses. The presence of both nuclear genomes in the hybrids was confirmed by the *Alu*I polymorphism of the PCR D1/D2 domain products (Fig. S1). The ability to grow on YPGE plates demonstrated the presence of complete mtDNA. In addition, all hybrids were excellent sporulants, but the viability of their spore was low, from 2 to 14%. Eleven F1 hybrids, six from *S. paradoxus* CBS 432 × *S. cerevisiae* CCY 21-4-96 crosses and five from *S. paradoxus* CBS 2908 × *S. cerevisiae* CCY 21-4-96 crosses, were selected for further analysis. The presence of both parental chromosome sets in all F1 hybrids was confirmed by the karyotype analysis using pulsed field gel electrophoresis (PFGE) (Fig. S2).

### Analysis of mitochondrial DNA (mtDNA)

**Figure 1:**
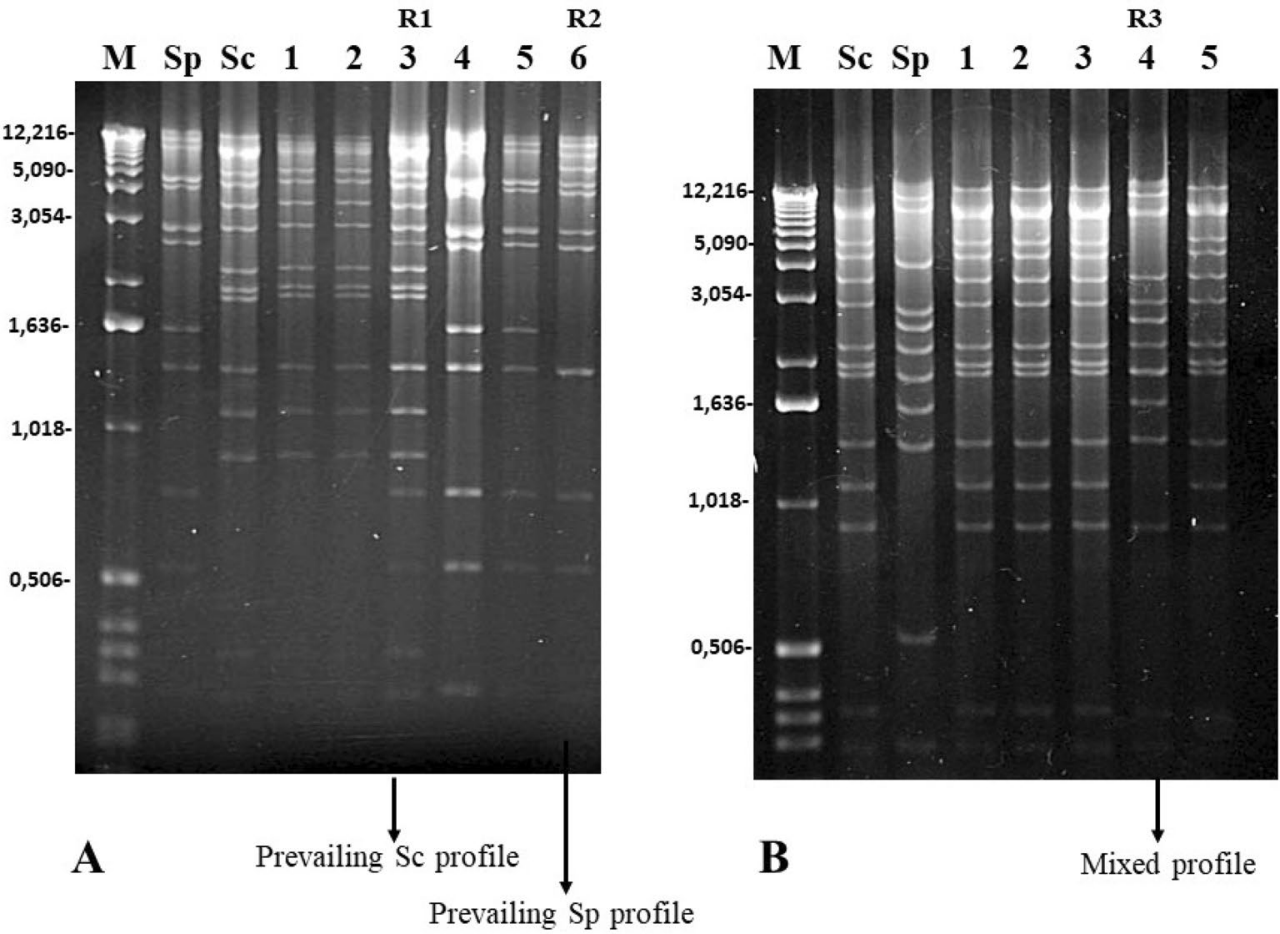
*EcoR*V restriction analysis of mitochondrial DNA (mtDNA) from the parental strains and the inter-specific hybrids. (**A**) Parental strains *S. cerevisiae* CCY 21–4-96 and *S. paradoxus* CBS 432, and their hybrids (1–6). (**B**) Parental strains *S. cerevisiae* CCY 21–4-96 and *S. paradoxus* CBS 2908 and their hybrids (1–5). Recombined *EcoR*V profiles are marked as Ry.

Restriction endonuclease analysis of mtDNA is widely used to distinguish between different yeast strains^32,33^. The *S. cerevisiae* and *S. paradoxus* mitochondrial gene orders differ from each other by two rearrangements, and they can be distinguished by *HinfI* or *EcoRV* digest^32,34^. mtDNA from 11 hybrids was subjected to *EcoR*V restriction analysis. The obtained patterns were compared for those of the parental strains (Fig. 1). Four hybrids originating from *S. cerevisiae* CCY 21-4-96 and the *S. paradoxus* strain CBS 432 showed mtDNA restriction patterns identical to those exhibited by the parental strains (Fig. 1A). However, two hybrids showed mixed restriction profiles (recombination rate 33.3%, hybrids 3-R1 and 6-R2; Fig. 1A). The 3-R1 mtDNA exhibited the same pattern as *S. paradoxus* CBS 432, but it contained some additional bands from *S. cerevisiae* CCY 21-4-96. The 6-R2 pattern showed a majority of bands identical to those of *S. cerevisiae* CCY 21-4-96, with one or two new larger bands and at least one band absent.

Four of the *S. cerevisiae* CCY 21-4-96 × *S. paradoxus* CBS 2908 hybrids acquired *S. cerevisiae* CCY 21-4-96 mtDNA, as can be inferred from their electrophoretic profiles (hybrids 1, 2, 3, and 5; Fig. 1B). Only one hybrid, 4-R3 (72 K), exhibited a mixture of bands belonging to both parents, with some parental bands missing (recombination rate 20.0%).

### Rearranged mtDNA forms are stable

To exclude a possibility of heteroplasmic mtDNA populations present in zygotes with mixed mtDNA profiles, the hybrids were grown for approximately 20 generations, and mtDNA from 10 single colonies was purified and subjected to *HinfI* or *EcoRV* restriction analysis. All recombined mtDNAs were stable since their unchanged restriction profiles were found in all single colonies (Figs. S3, S4).

### Characterization of mtDNA rearrangements

**Figure 2:**
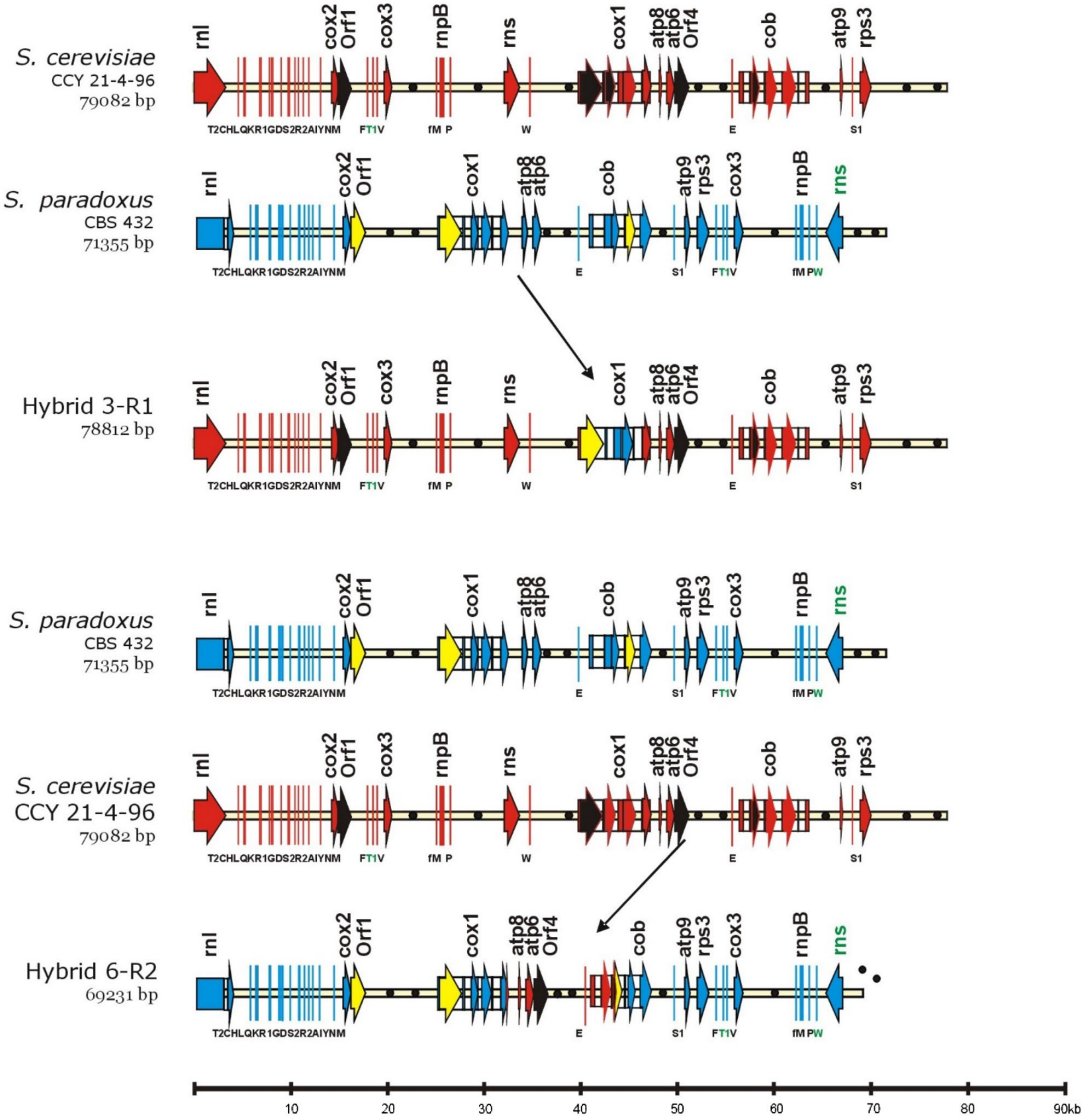
The genetic organization of recombined mtDNA in hybrids. For simpler comparison, the circular genomes, exported from Vector NTI, were aligned at the beginning of the large rRNA subunit (rnl). Genes are marked with colored rectangles with arrows or lines, and origins of replication are marked with black circles. The RNA of the rnpB gene and tRNA genes are indicated by thin lines. Introns are marked with white rectangles. Gene nomenclature follows the rules described in GOBASE (atp for ATP synthetase subunits; cox for cytochrome oxidase subunits; cob for cytochrome b; rns for small rRNA ribosomal subunit; rnl for large rRNA ribosomal subunit; T2, C, H, etc. for particular tRNA coding genes; rps3 for ribosomal protein; and rnpB for the RNA subunit of RNase P). Sections originating from *S. cerevisiae* are marked in red, and those originating from *S. paradoxus* are in blue. Introns and free standing ORFs known as mobile, in *S. cerevisiae* are marked in black^35^. Their homologs in *S. paradoxus* are marked in yellow. Sizes are given on the bottom line in kbp. GenBank accession numbers MW367979, JQ862335, MW367976, MW367977.

**Figure 3:**
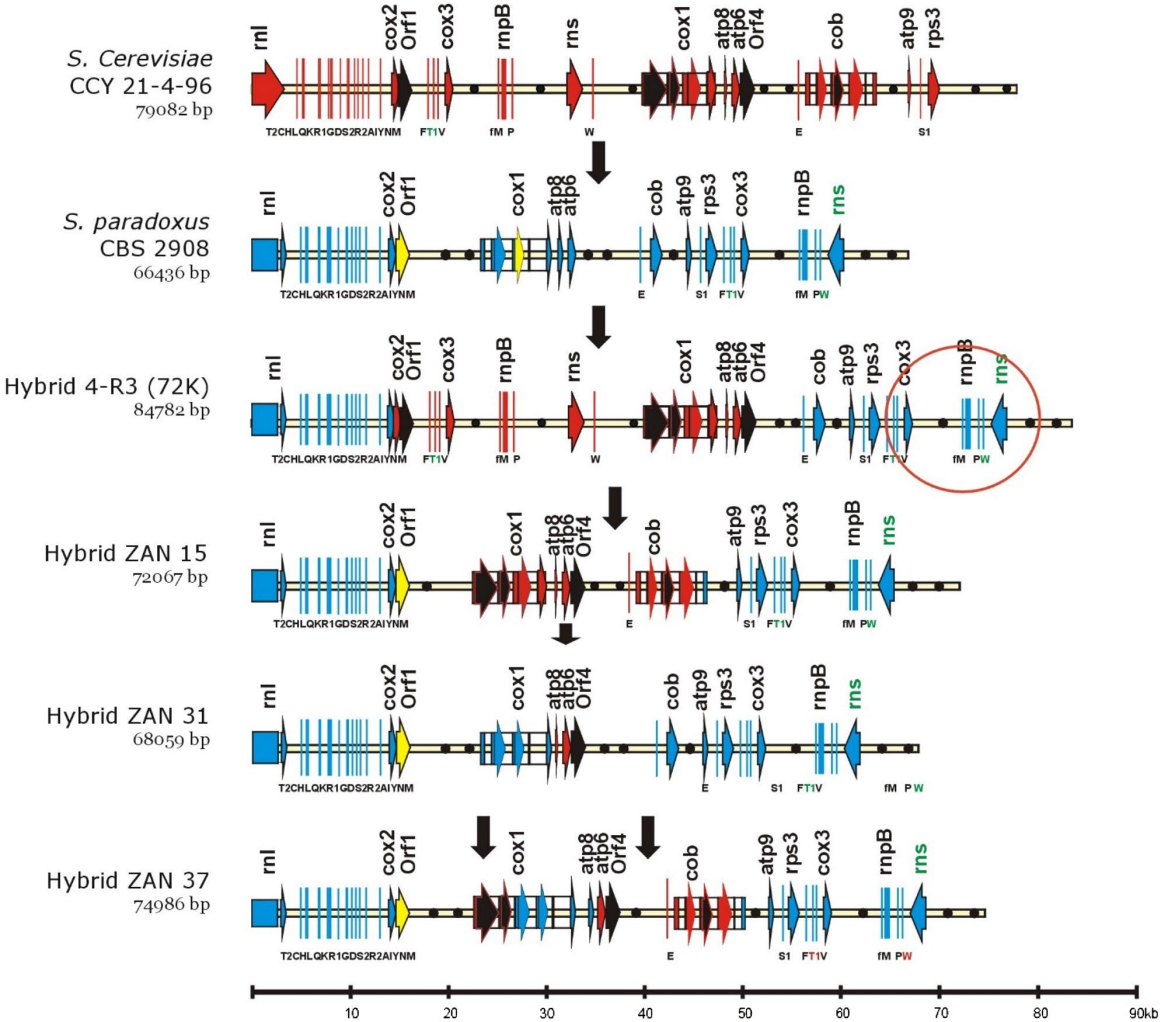
The genetic organization of recombined mtDNA in hybrids. For simpler comparison, the circular genomes, exported from Vector NTI, were aligned at the beginning of the large rRNA subunit (*rnl*). The gene legend is the same as in Fig. 2. Sections originating from *S. cerevisiae* are marked in red and those originating from *S. paradoxus* are marked in blue. Introns and free standing ORFs known as mobile, in *S. cerevisiae* are marked in black^35^. Their homologs in *S. paradoxus* are marked in yellow. Sizes are given on the bottom line in kbp. GenBank accession numbers MW36797, KX657748, MW367978, MW367980-2.

To determine the exact organization of the rearranged mitochondrial molecules, purified mtDNAs were directly sequenced with primers specific to the conserved regions of several mitochondrial genes (*cox1*, *cox2*, *cox3*, *cob*, *rns*, *rnl*, *atp9*, *trnM*). However recent advances in the assembly of mtDNA sequenced by Illumina MiSeq^34^ allowed us to obtain complete sequences of recombined mitochondrial genomes (Table S1) that were confirmed by RFLP. Generally, the rearranged molecules were composed of a major skeleton from one parental molecule with small regions substituted with segments from another parental molecule (Figs. 2, 3). This observation suggested that recombination between two mtDNA molecules had occurred.

### Hybrid 3-R1 molecule

The original hybrid 3-R1 and stable subclones contained all genes from *S. cerevisiae*, except for *cox1* (Fig. 2). The *cox1* gene was recombined and had a 6600 bp segment spanning *S. paradoxus Spcox1*I2–*Spcox1*I5γ with 5′ and 3′ flanking exon regions.

### Hybrid 6-R2 molecule

The recombined 6-R2 mtDNA molecule received a majority of genes from *S. paradoxus* (Fig. 2), but three genes, *atp6*, *atp8*, and *trnE*, were introgressed from *S. cerevisiae*. The recombination sites were identified in the *cob* and *cox1* genes. The 5′ junction was located in the last exon of the *cox1* gene, and the 3′ junction was located in the *cob* gene in the *Spcob-*I3 intron that is homologous to *Sccob-*I2 intron (Fig. 2).

### Hybrid 4-R3 (72 K) molecule

The completed mitochondrial genome of 4-R3 (72 K) confirmed that the mtDNA molecule was recombined and consisted of sequences originating from both parents. Sequences of the genes *atp6*, *atp8*, *cox1*, *rns*, *rnpB*, and *cox3* were identical to those of *S. cerevisiae* CCY 21-4-96, and sequences of genes *rns*, *rnpB*, *cox3*, *trn*, *rnl*, *var1*, *atp9*, and *cob* were identical to those of *S. paradoxus* CBS 2908.

The consequence of this recombination was duplication of *trnF*, *trnT*, *trnV*, *cox3*, *trnM*, *rnpB*, *trnP*, *rns*, and *trnW* genes. Recombination sites were identified at *cox2* (positions 531–570 from ATG) and downstream of *ori4 S. paradoxus* (positions 204–107) (Fig. 3).

**Figure 4:**
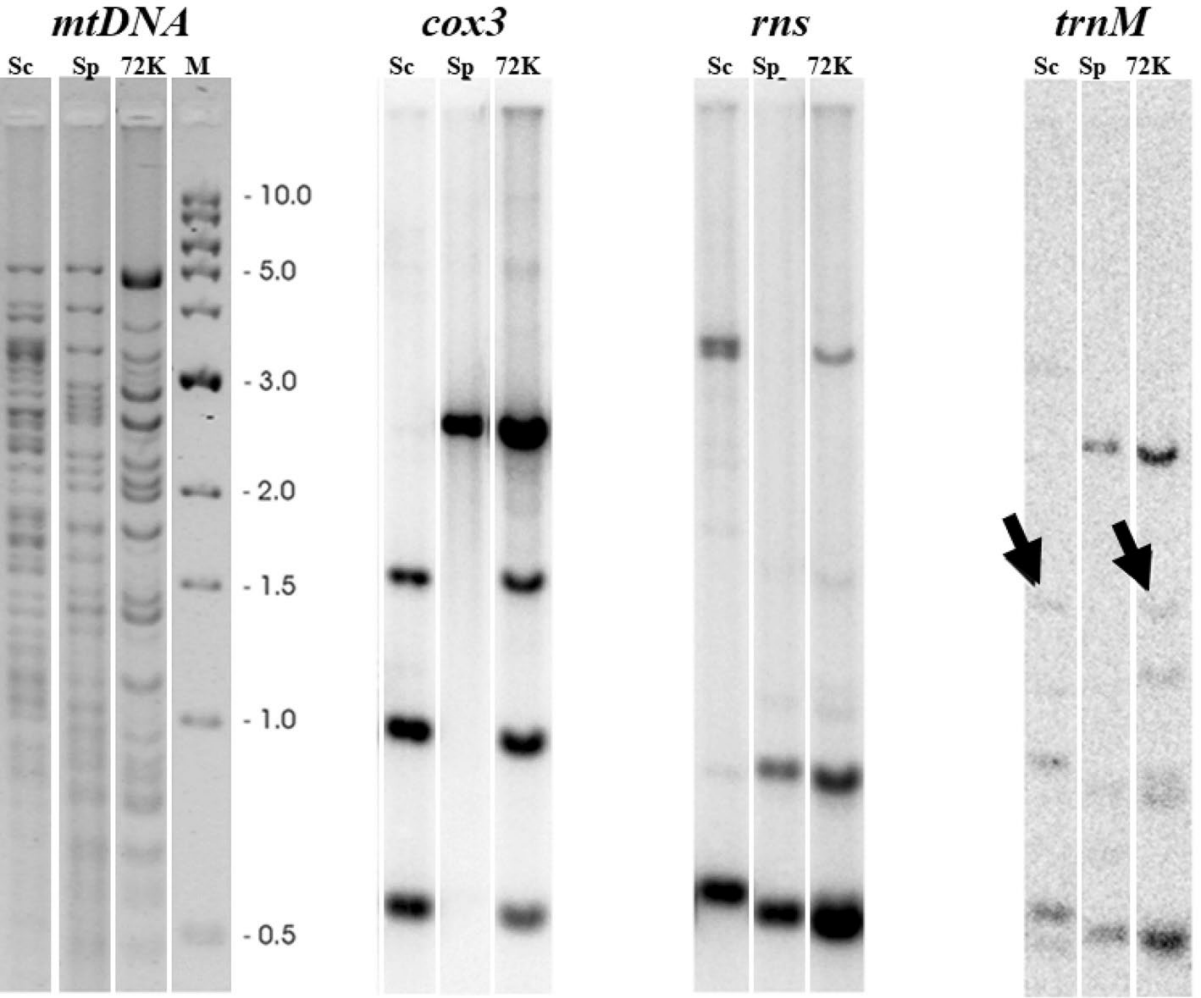
Southern blot analysis of mtDNA molecules from the parental strains *S. cerevisiae* CCY 21-4-96 and *S. paradoxus* CBS 2908 and hybrid 4-R3 (72 K). *HinfI* restriction analysis of mtDNAs is at the left, while the autoradiograms showing hybridizations with the *cox3*, *rns*, and *trnM* probes are to the right. The *cox3* and *rns* probes were ^32^P-labeled PCR products of the corresponding genes. The *trnM* probe was an oligonucleotide of which the 5′ end was labeled with γ^32^P-ATP. The arrows on the *trnM* picture show the *S. cerevisiae* band that is present in the 4-R3 (72 K) hybrid.

The duplication of *rns*, *cox3*, and *trnM* (adjacent to *rnpB*) genes was also confirmed by Southern blot analysis (Fig. 4). The *HinfI* restriction pattern of the 4-R3 (72 K) mtDNA molecule was hybridized with radioactively labeled probes and compared to those of the parental strains. All three probes were hybridized to combination of parental bands in the restriction pattern of the 4-R3 (72 K) molecule.

### How frequent is duplication in mtDNA?

We addressed the following question. At what frequency do mtDNA variants with duplicate regions arise? Selection based on colony appearance (smooth colonies) and the ability to grow at 37 °C provides only a limited number of hybrids. To analyze multiple recombined mitochondrial genomes, the mtDNA from *S. cerevisiae* CCY 21-4-96 was transferred via cytoduction to the auxotrophic laboratory strain 3C with geneticin resistance resulting in the strain 3C-CCY. *S. cerevisiae* 3C-CCY/*S. paradoxus* CBS 2908 hybrids were then selected on minimal medium with geneticin, and a larger set of sixty hybrids was analyzed. Although some recombined mtDNA were found, none of them contained the restriction profile typical for 4-R3 (72 K) (Fig. S5). Apparently, gene duplication in mtDNA is a rare event. To identify other recombination variants in hybrids, we sequenced three complete recombined mitochondrial genomes of hybrids (ZAN) by Illumina MiSeq. All recombined molecules are the result of transposition from *S. cerevisiae* to *S. paradoxus* (Figs. 3, 4). The ZAN15 molecule received about a 23,840 kb segment from the beginning of *cox1* up to the end of the *cob* gene. The precise recombination sites were at the beginning of the first exon of *cox1* and the last exon of the *cob* gene. The ZAN31 molecule contains about 3280 bp long DNA containing *ORF4* and *atp*6 from *S. cerevisiae.* The recombination sites were mapped in the region 251–332 downstream the *atp*6 start codon. The mtDNA in the ZAN37 hybrid results from two recombination events. The first one was triggered by the homing and co-conversion of flanking exons. The recombination sites were identified in the flanking exons. The second recombination event was also initiated by *ORF4*, and due to the mobility of *cob-*I2 it is about 14,760 bp long. The recombination sites were mapped downstream of the *atp*6 start codon and at the beginning of the last *cob* exon.

## Discussion

In *Saccharomyces* yeasts, the diverse and highly reticulated mtDNAs show signatures of recombination and horizontal gene transfer within and between species^27–30,36–38^. To understand mtDNA transmission and the rate of recombination in interspecific *Saccharomyces* hybrids, we studied the progeny from the mating of two very closely related species: *S. cerevisiae* and *S. paradoxus.* The gene order of the *S. paradoxus* mitochondrial genome (European lineage) differs from that of *S. cerevisiae* by only two rearrangements and is well preserved within the species. In addition, there is wide variability in intron content and intergenic sequences, even among isolates of the same species^28,34,36,39,40^. To avoid any modification due to auxotrophic mutations, hybrids were selected according to the ability to grow at 37 °C (*S. cerevisiae*) and the production of rough colonies (*S. paradoxus*). This selection should favor the transmission of *S. cerevisiae* mtDNA because mitochondria-encoded genes from other *Saccharomyces* contribute to the cold tolerance in yeast hybrids^41–43^. However from 11 *S. cerevisiae* × *S. paradoxus* hybrids, two inherited mtDNA from *S. paradoxus* and six inherited mtDNA from *S. cerevisiae*, and in three hybrids we found mixed mtDNA restriction patterns, indicating recombination events. These hybrids were homoplasmic as the single colonies, and after approximately 20 generations, they provide the same restriction profiles. The frequency of recombination inferred from the restriction analysis of 60 *S. cerevisiae* 3C-CCY/*S. paradoxus* CBS 2908 hybrids was 15% (Fig. S5, Table S2).

The exact organization of the rearranged mitochondrial molecules was determined by genome-wide sequencing, and the recombination sites were confirmed by primer walking sequencing. All rearranged molecules were composed of a major skeleton from one parental molecule, with small regions introgressed from the second parental mtDNA. Sequence differences between interspecific mtDNA molecules allowed us to precisely map the recombination spot within the 16–200 bp region (Fig. S6).

Specific mtDNA regions play a crucial role in recombination, namely mobile introns^44–46^ and GC clusters^34,47^, where the insertion sites act as recombination "hot spots". Generally, the mobilization of group I (GI) introns coding open reading frame (ORFs) and free-standing ORFs are initiated by double-strand break formation in an intronless gene or ORF-free allele^15,45,48^. The intron or ORF-coded endonuclease (HOE) cleaves DNA, generating DNA ends that invade the homologous exon or ORF-lacking sequences of an element-containing allele. The process is called homing and is accompanied by the co-conversion of flanking sequences. Group II (GII) introns also possess a reverse-transcription-independent homing pathway that is initiated by the intron-encoded endonuclease (cleaves the antisense strand) and completed by the double-strand break repair (DSBR) recombination system of yeast mitochondria^44,49^. In addition, there is wide variability in intron content and intergenic sequences, even among isolates of the same species^28,34,36,39,40^. Therefore, if two isolates having different intron composition should mate, the homing/transposition pathway of numerous introns is potentially initiated. It was believed that a consequence of this process should be strong activation of the recombination system, associated with replication and repair machinery^26,50,51^. However, the role of repair machinery is questionable because the absence of certain genes from this group (*NTG1*, *MGT1*) does not affect the frequency of mtDNA recombination^26^.

**Figure 5:**
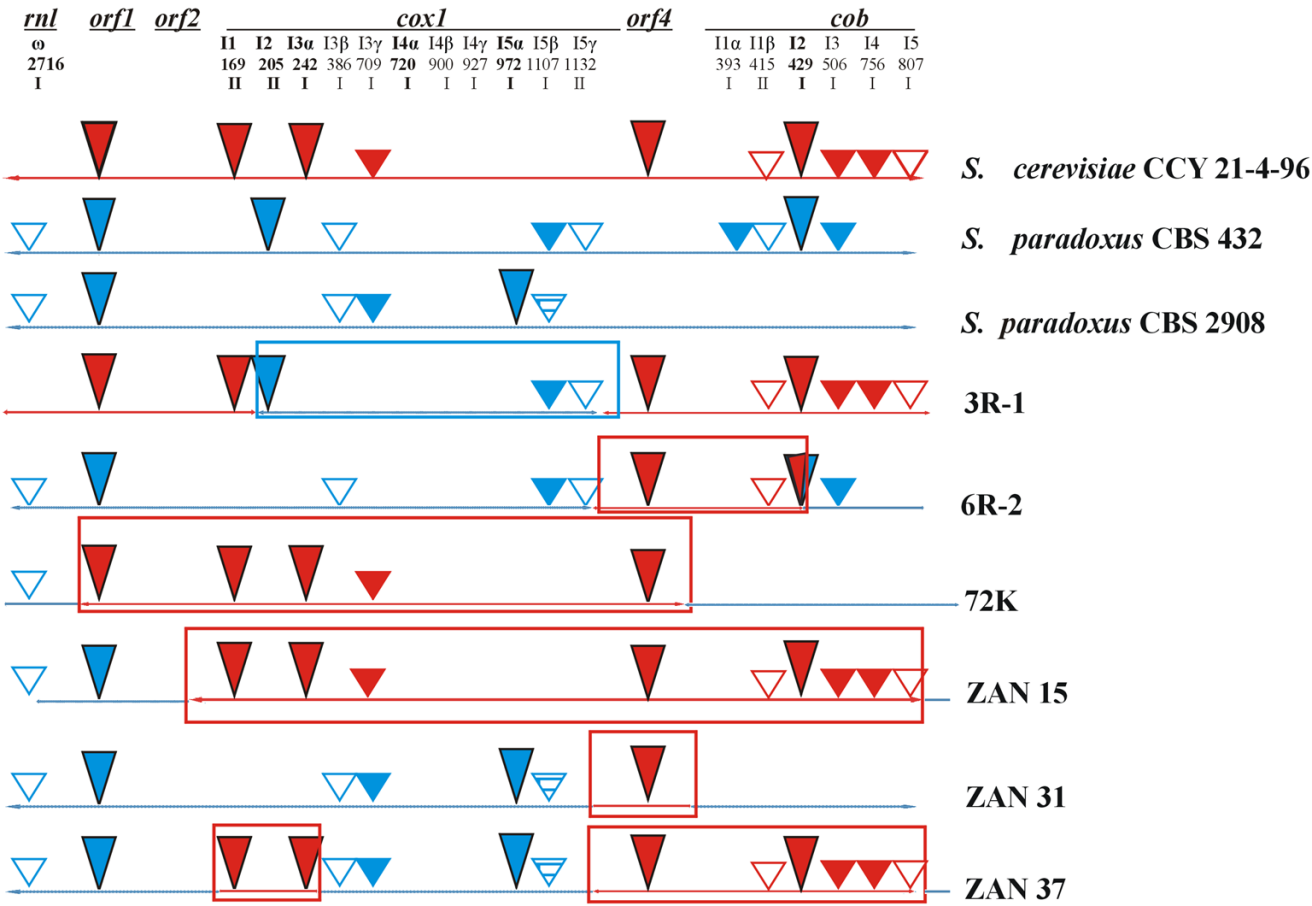
Occurrence of introns and free-standing ORFs in hybrids and parental yeasts. Sections originating from *S. cerevisiae* are marked in red and those originating from *S. paradoxus* are marked in blue. Introns and free standing ORFs known as mobile, in *S. cerevisiae* and their homologs in *S. paradoxus* are filled long triangles^35^. Introns and free-standing ORFs with at least one motif characteristic for the homing endonucleases (LAGLIDADG, GIY-YIG, etc.) are filled small triangles. Introns without reading frame are white; with truncated ORF interrupted by stop codon are striped. Numbers indicate the base preceding the intron insertion site in the CDS. Positions of introns, known as mobile, in *S. cerevisiae* are marked in bold; I—group I introns, II—group II introns. *ORF1, ORF4* free standing open reading frames. Introgressed parts are in rectangles.

In *S. cerevisiae* mtDNA three genes (*rnl, cox1,cob*) are interrupted by introns. Among these (GII) I1 and I2, and group I introns (GI) I3α, I4α and I5α in *cox1* are known to be mobile^34,35,52,53^. In many strains they are inactive due to the interruption with GC clusters or mutations introducing premature stop codons^28,34,35^. In *cob* gene GI I2, I3 and I4 introns code for maturases and only in some strains I2 might be mobile, owing to only 2 amino acid substitutions^34,35,54^. GI intron known as ω interrupts *rnl* gene and often contains “freestanding” open reading frame coding for I-*Sce*I endonuclease necessary for its mobility^35,55^. Free standing ORFs were found during early sequencing experiments according to LAGLI-DADG motif reminiscent to the intron coded ORFs. HOE-like reading frames were found downstream *cox2* gene continuous open reading *ORF1*, downstream *cox3 ORF2* and *ORF4*, residing at the 3′ end of the *atp6* gene (Fig. 5)^34,35^. Product of *ORF4* (also referred to as *ENS2*), is the subunit of Endo.*Sce*I endonuclease, whereas second subunit is coded in nucleus and imported to the mitochondria.

The occurrence of mobile elements near many recombination junctions suggests causal relationship. Five out of 14 junctions are located in the *cox1* gene (Figs. 5, S6).

Three 5′ recombination junctions are in the sequence of exon 1 of the *cox1* gene and are apparently the result of homing of mobile introns *cox1*-I1 and *cox1*-I2. Two 5′ recombination sites are in the same position between *atp8* and *atp6* genes, resulting from the transplacement of the *ORF4* element. Most of the 3′ ends of novel recombination sites were found in the *cob* gene and are seemingly the result of the homing/transposition of the *cob*-I2. In summary, from seven identified recombination events in six different hybrid mtDNAs, three can be considered as the synergic result of the *ORF4* and *cob*-I2 transplacement. One can be attributed to cooperative transposition of *ORF4* with *cox1*-I, and the largest segment was mediated by their cooperative transfer with the *ORF1* element. The most plausible explanation of all recombination events is homing/transposition of mobile elements accompanied by the co-conversion of flanking sequences, which can be several kbp long^56,57^. Apparently, *ORF4* is the strongest element that triggers the recombination. *ORF4* (*ENS2*) codes for *Endo.Scel* endonuclease that cleaves mtDNA every 2–3 kb, but the main cleavage site is at the end of the *atp6* gene, allowing homing into the vacant site^58^. The main recombination site was in the region that was 700–900 bp upstream of the recognition site, seemingly due to co-conversion of flanking sequences. In addition, the recombination strength is elevated by the absence of *ORF4* in both *S. paradoxus* strains used for hybrid construction^34,39^. Evidence of *ORF4* involvement in the introgression of *atp6* has been observed in the study of complex *atp6* phylogeny^59^.

In one event, the recombination of two large segments—*trnE-cox2* from *S. paradoxus* and *ORF1-atp8* from *S. cerevisiae*—yields duplication of the genes for *cox3*, *rnpB*, and *rns* and six *tRNA*s. Duplications in the mitochondrial genome are relatively rare and have been observed predominantly in plants^60–62^. Large-scale duplications of human mitochondrial DNA were found associated with Kearns-Sayre syndrome^63^. Data concerning duplication in yeast are scarce. To our knowledge there is only one paper describing the duplication from Clark-Walker laboratory^64^. The reported strain with duplication did not exhibit any growth phenotype. The same we observed in the case of our hybrid with duplication.

A characteristic feature of duplicate sections in animal mtDNA is tandemly arranged repetitive sequences resulting from slipping of the synthesized slipped strand mispairing fiber^65–67^. Major events in the evolution of animals, such as multicellularity and the emergence of symmetry, are accompanied by changes in the organization of mtDNA^68^. The most profound feature of *Saccharomyces* species is the conservative and species-specific gene order in their mtDNAs^34^. In addition, they offer a great opportunity to study evolutionary processes experimentally. The process called “hybrid speciation” implies that hybridization has been involved in the origin of new species^69,70^. All models of gene reordering in mtDNA consider the formation of duplicate regions^66,67^. A tandem duplication followed by random gene loss (TDRL model) is the most important mechanism of gene order rearrangements in mitochondrial genomes^68,71,72^. Consequently, the duplication of mtDNA may lead to changes in the gene order in the hybrid progeny and accompany the speciation process. Groth et al.^73^ already proposed that a change in the mitochondrial gene order may be a step in sexual isolation and the generation of new species. If two isolates having different gene orders should mate, homologous recombination in the zygote would create a number of mtDNA molecules that would lack a complete set of mitochondrial genes or contain a gross duplication. Indeed, in our study when *S. cerevisiae* and *S. paradoxus*, having a different mitochondrial gene order, were crossed, a rare genome with the duplication of several genes arose, which can be used to test this hypothesis.

Duplication and recombination are mediated by the translocation of selfish mobile elements. Their extent of benefit and harmfulness for the host is still discussed^74^. Selfish mitochondrial introns, as well as free-standing ORFs, are beneficial to the host if the mother molecule is challenged with another molecule for transmission to the progeny. Mobile elements trigger mtDNA recombination in this situation, ensuring the transfer of adjacent exons, often large regions, into the progeny of recombinant molecules.

## Methods

### Yeast strains and media

The following *Saccharomyces* strains were used in our studies: *S. cerevisiae* CBS 1171, *S. cerevisiae* CCY 21-4-96, *S.* *paradoxus* CBS 432, and *S. paradoxus* CBS 2908. CBS isolates were from the Centraalbureau voor Schimmelcultures, Delft, the Netherlands. The CCY isolate was from Culture Collection of Yeasts, Institute of Chemistry, Slovak Academy of Sciences, Bratislava, Slovakia. Strain 3C-CCY (*MATα*, *leu2Δ0*, *his3Δ1*, *ura3Δ0*, *ma15Δ0*, *Δaac1*: *kanMX4*; mtDNA from CCY 21-4-96) was prepared by cytoduction according to other work^32^. Yeasts were grown overnight on YPD medium (1% yeast extract, 2% bacto peptone, and 2% glucose) or YPGE (1% bacto peptone, 1% yeast extract, 3% glycerol, 2% ethanol) at 28 °C.

### Hybrid preparation

*Saccharomyces paradoxus* CBS 432 and CBS 2908 strains do not grow at 37 °C and produce smooth colonies. The *S. cerevisiae* CCY 21-4-96 strain grows at 37 °C and produces rough colonies. Homothallic diploid *S. cerevisiae* CCY 21-4-96, *S. paradoxus* CBS 432, and CBS 2908 strains were starved for 3–5 days in 1% potassium acetate media at room temperature to induce sporulation. The asci were digested by Zymolyase (0.5 mg/ml) for 30 min at 30 °C, and individual spores were crossed by micromanipulation. Smooth colonies, which were able to grow at 37 °C, were isolated. *S. cerevisiae* W303 *MAT alpha ade2 ura3 leu2 trp1 his3* haploid strain was crossed with *S. paradoxus* CBS 2908 spores and prototrophic colonies able to grow at 37 °C were isolated.

### Tetrad dissection

Sporulation was induced for 3–5 days at room temperature on solid 1% potassium acetate media, and spores were dissected by micromanipulation.

### Chromosome separation

Chromosomes of putative hybrids and parental strains were isolated and separated by PFGE employing a CHEF Mapper XA (Bio-Rad) as previously described^13^.

### Restriction analysis of 26S rRNA

Total DNA was extracted from each hybrid and parental strain^75^. The D1/D2 domain of the 26S rRNA gene was amplified by PCR using primers NL1 and NL4^76^. The PCR products were digested to completion with *Alu*I restriction enzyme. The restriction fragments were resolved on 1% agarose gel.

### mtDNA purification, restriction analyses, sequencing, assembly, and annotation

mtDNA was purified by bisbenzimide/CsCl buoyant density centrifugation^77^ or by differential centrifugation^78^. Restriction analyses of mtDNA were performed using *EcoR*V and *Hinf*I restriction enzymes^32^. Whole genome Illumina MiSeq sequencing and assembly was performed as previously described^34^. Briefly, paired reads were trimmed and assembled into individual contigs using CLC Genomics Workbench 9.5 (Qiagen, Hilden, DE). Contigs containing mtDNA were selected by comparison (BLASTN) with the known mtDNA from *S. cerevisiae* and *S. paradoxus* and assembled into a single molecule using the Vector NTI v.9.0 (v.10) software package from InforMax, Inc. Gene annotation was carried out using MFannot (http://megasun.bch.umontreal.ca/cgi-bin/mfannot/mfannotInterface.pl) as described previously^34^. Novel junctions were sequenced directly by dye terminator sequencing chemistry with a Genetic Analyzer (Applied Biosystems, Foster City, CA, USA) (ABI310 and ABI3100-Avant) as described in other work^39^.

### Southern blot analysis

CsCl-purified mtDNAs digested with *Hinf*1 restriction endonuclease were analyzed by standard gel electrophoresis through 1% agarose gel at 2.0 V/cm in 1% TBE buffer. The DNA in the gel was denatured (1.5 M NaCl, 0.5 M NaOH) for 30 min, neutralized (1.5 M NaCl, 1 M Tris–HCl, pH 7.5) for 15 min, and transferred to a Hybond™-N^+^ membrane (GE Healthcare) in 20 × SSC (1.5 M NaCl, 0.15 M sodium citrate) for 2 h by vacuum blotting (VacuGene^TM^XL). Finally, the membrane was washed in ddH_2_O, and DNA was fixed by UV light. *Cox3* and *rns* genes were detected by Southern blot analysis using ^32^P-labeled PCR products as probes (GE Healthcare). PCR products were purified with the QIAquick PCR extraction kit (Qiagen, Dorking, UK). Unincorporated nucleotides were removed by gel filtration through G-25 columns (GE Healthcare). After prehybridization, the membrane was hybridized (0.25 M Na_2_HPO_4_, 7% SDS, 1 mM EDTA) at 60 °C for 12 h. The membrane was washed twice at room temperature for 5 min and once at 60 °C for 20 min with 2% SDS and 100 mM Na_2_HPO_4_. The membrane was stripped (0.4 M NaOH) for 2 h at 40 °C and re-hybridized more than once. The *trnM* gene adjacent to the *rnpB* gene was detected using the Fmet oligonucleotide probe, of which the 5′ end was labeled with γ^32^P-ATP by a T4 polynucleotide kinase (ABgene). The membrane was pre-hybridized and hybridized as described above at 40 °C and washed twice at room temperature for 10 min. Signals were detected using Phospho-Screen (imaging Screen-K, 32*43 cm, catalog #170-7841, Bio-Rad) and Personal Imager Fx (Bio-Rad).

## Supplementary Information

Supplementary Information.
