# Supplementary material for: Mitochondrial DNA duplication, recombination, and introgression during interspecific hybridization
Source: Sci Rep. 2021 Jun 16;11:12726. doi: 10.1038/s41598-021-92125-y (PMC8209160; doi:10.1038/s41598-021-92125-y)
Supplement: Supplementary file 1 — Supplementary Information. [file 41598_2021_92125_MOESM1_ESM.docx]

Supplementary Information for

**Mitochondrial DNA Duplication, Recombination, and Introgression During Interspecific Hybridization**

Silvia Bágeľová Poláková *et al*.

Correspondence to: pavol.sulo@uniba.sk

**This PDF file includes:**

**Supplementary tables**

**Table S1**

**Table S2**

**Supplementary figures**

**Fig. S1**

**Fig. S2**

**Fig. S3**

**Fig. S4**

**Fig. S5**

**Fig. S6**

**Table S1**  **Sequencing summary**

| **Strain** | **Paired reads** | **Average**  **length** | **^a^Coverage**  **X** | **Genbank**  **number** | **Contigs/**  **nts** | **Coverage**  **%** | **mtDNA**  **size** |
| --- | --- | --- | --- | --- | --- | --- | --- |
| *S. cerevisiae*  CCY 21-4-96 | 1,075,526 | 277 | 3767 | MW367979 | 8/75158 | 95.9 | 79082 |
| 3-R1 | 7,087,029 | 173 | 15557 | MW367976 | 5/78212 | 99.3 | 78812 |
| 6-R2 | 2,480,795 | 175 | 3014 | MW367977 | 4/69105 | 99.8 | 69231 |
| 72K | 1,108,002 | 145 | 6270 | MW367978 | 2/84210 | 99.3 | 84782 |
| ZAN15 | 5,783,394 | 168 | 13482 | MW367980 | 5/71287 | 99.3 | 72067 |
| ZAN31 | 1,282,832 | 239 | 4504 | MW367981 | 3/66998 | 98,4 | 68059 |
| ZAN37 | 1,377,866 | 263 | 4838 | MW367982 | 2/73930 | 98.7 | 74896 |

^a^Coverage calculated as *LN*/*G*, where *L* is the read length, *N* is the number of reads and *G* is the mitochondrial genome length

**Table S2 The occurrence of mtDNA types in**

***S. paradoxus*/*S. cerevisiae* interspecific hybrids**

| **mtDNA**  **restriction profile** | **Number of hybrids** | **Occurrence**  **in %** |
| --- | --- | --- |
| *S. paradoxus* CBS 2908 | 1 | 1.7 |
| *S. cerevisiae* 3C-CCY | 50 | 83.3 |
| 72K | 0 | 0 |
| ZAN15 | 2 | 3.3 |
| ZAN31 | 2 | 3.3 |
| ZAN37 | 2 | 3.3 |
| Other recombined | 3 | 5.0 |

**Fig. S1.** *Alu*I restriction fragment polymorphism of a PCR product of the D1/D2 domain. **A)** Parental strains. **B)** Putative hybrids.

**Fig. S2.** Pulse field gel electrophoresis of the intact chromosomes from hybrids marked as Hx and original parental strains from which hybrids were derived. **A)** Parental strains *S. paradoxus*^T^ CBS 432, *S. cerevisiae* CBS 1171 and their hybrids. **B)** Parental strains *S. paradoxus*^T^ CBS 2908*,* *S. cerevisiae* CBS 1171and their hybrids.


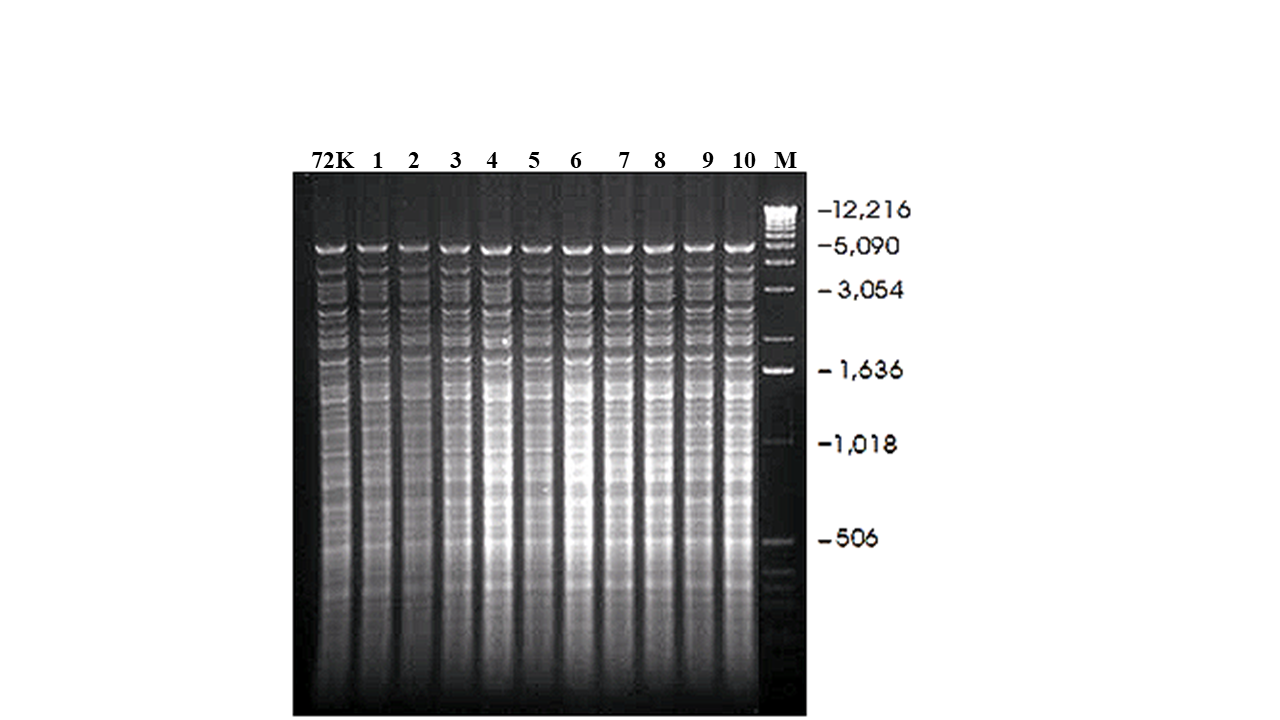


**Fig. S3. Mitotic stability of 4-R3 (72K)**

*Hinf*I restriction analysis of mtDNA from original hybrid and its single colonies propagated during the vegetative phase. 1 - original hybrid 4-R3 (72K), 2-11 – single colonies propa1gated mitotically, 12 - DNA marker in kb

**Fig. S4. Mitotic stability of 3-R1**


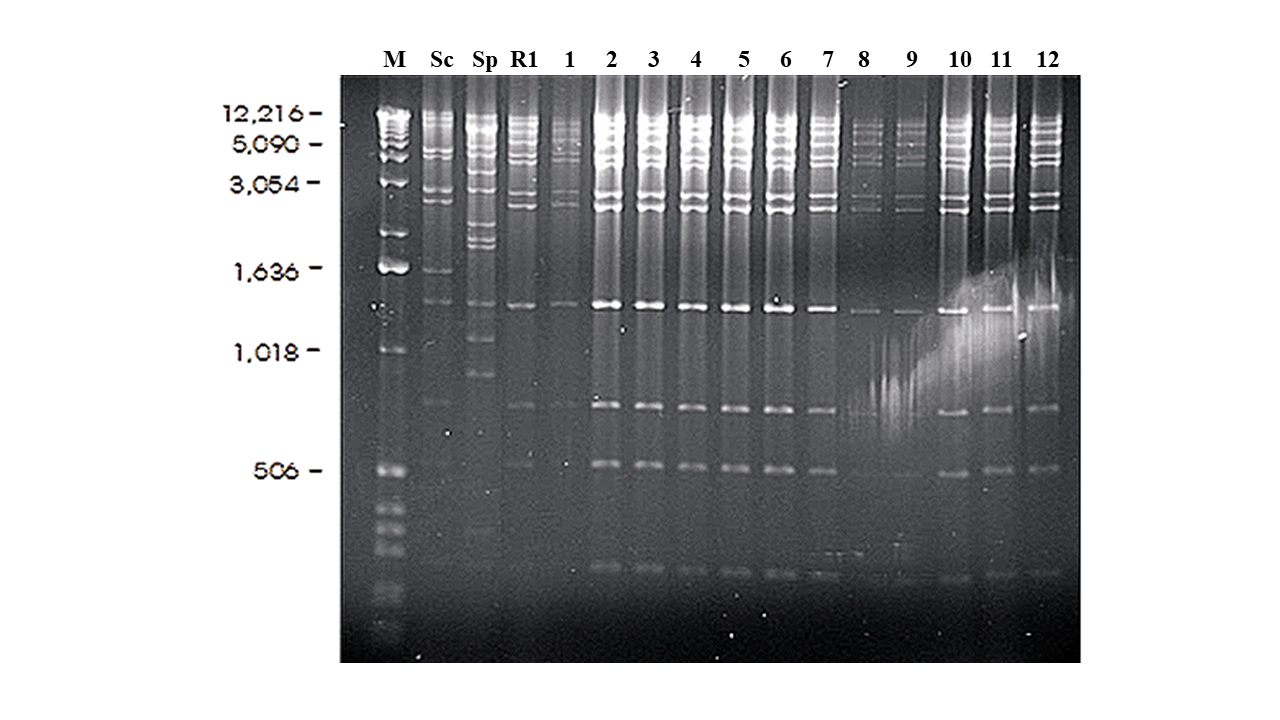


*EcoRV* restriction analysis of mtDNA from original hybrid and its single colonies propagated during the vegetative phase. 1 - original hybrid 3-R1, 2-11 – single colonies propagated mitotically, 12 - DNA marker in kb

**A**. **B.**

**M Sc 72K Sp 55 37 15**

**C.**

**Fig. S5. *Hinf*I** **restriction analysis of mtDNA from the parental strains and the inter-specific hybrids. Sp** *S. paradoxus* CBS 2908; **72K** *S. cerevisiae*/*S. paradoxus* hybrid with duplication; **Sc** *S. cerevisiae* strain 3C-CCY (with mtDNA *S. cerevisiae* CCY 21-4-96), 1-10 interspecific hybrids. **M** size standard λ/*Pst*I. Recombined mtDNA in hybrids **A.** lines 3, 8; **B**. line 9; **C.** lines 55, 37, 15. Restriction profiles for hybrids ZAN15 – 15; ZAN37 – 37 panel **C**; ZAN31 – line 9 panel **B**.


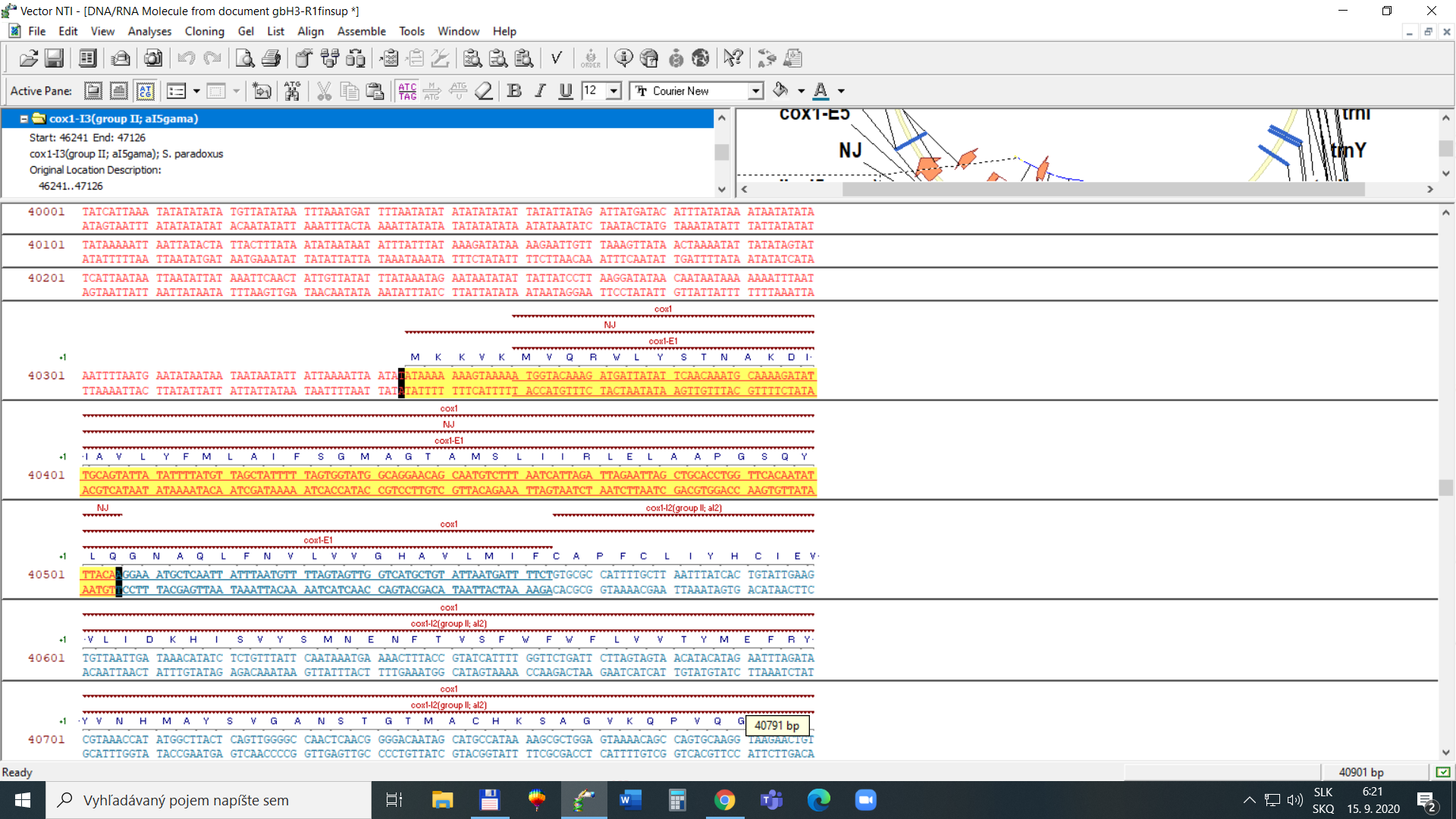


**5´junction**


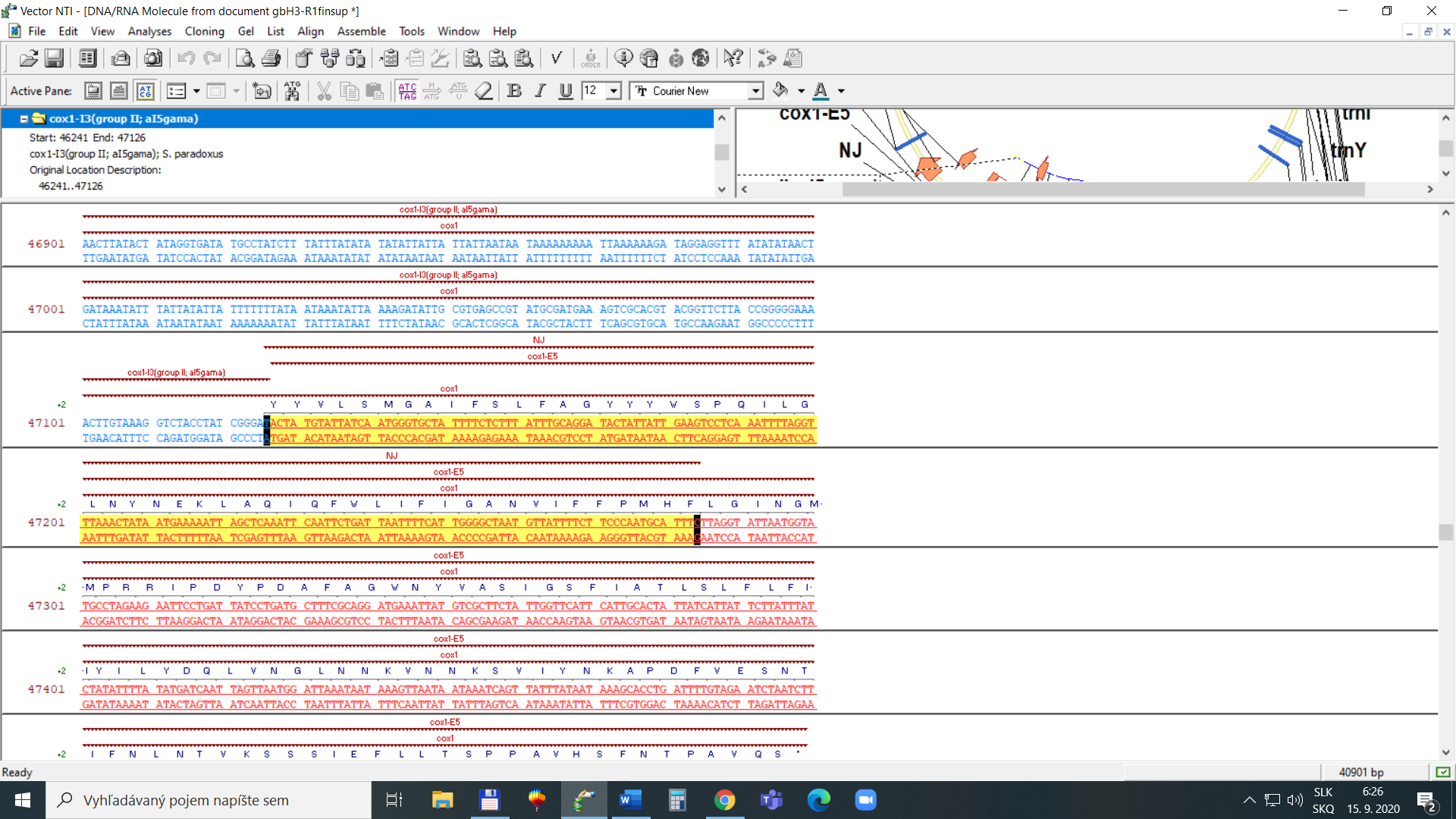


**3´junction**

**A. Hybrid 3-R1 molecule**


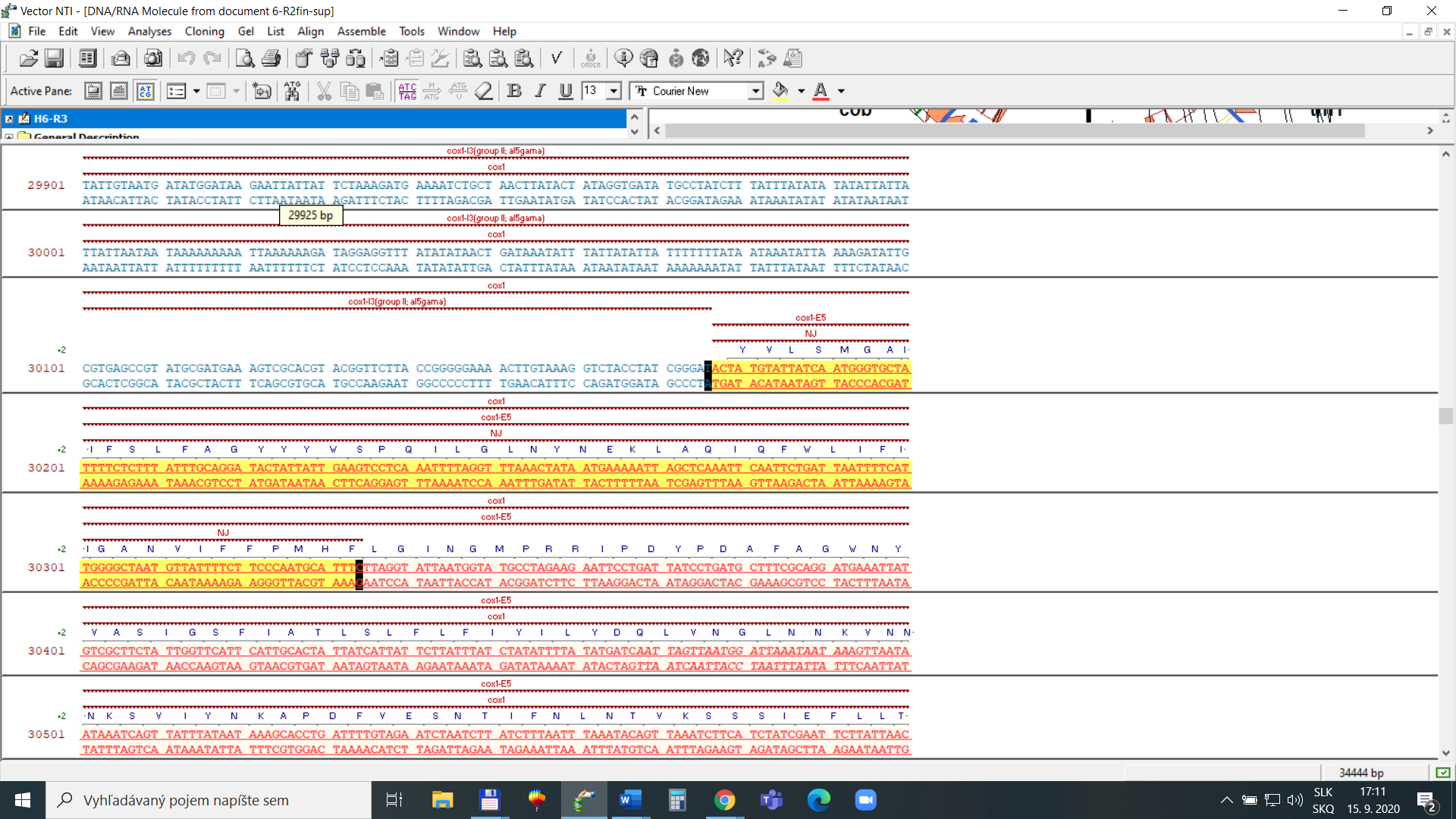


**5´junction**


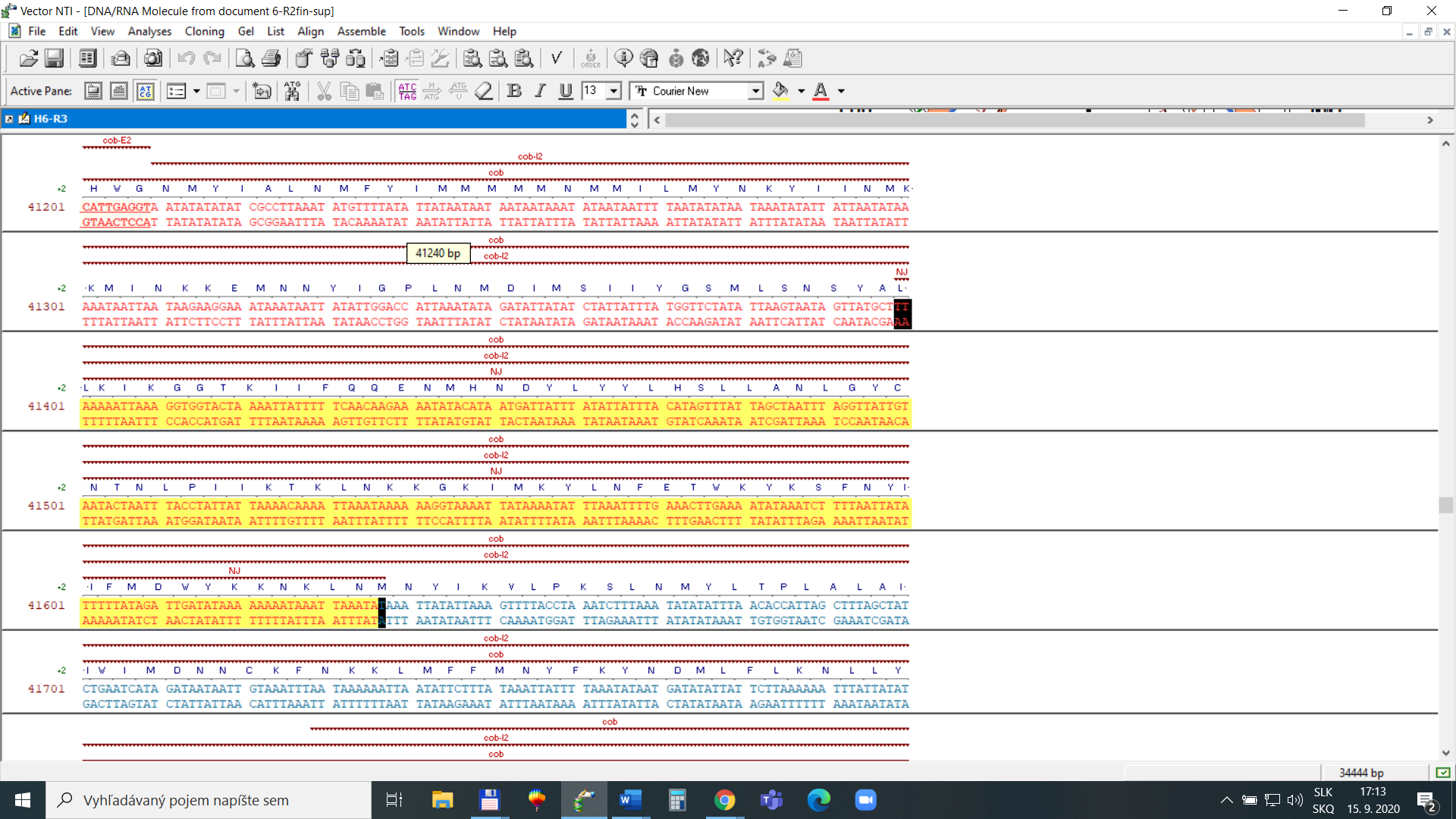


**3´junction**

**B. Hybrid 6-R2 molecule**


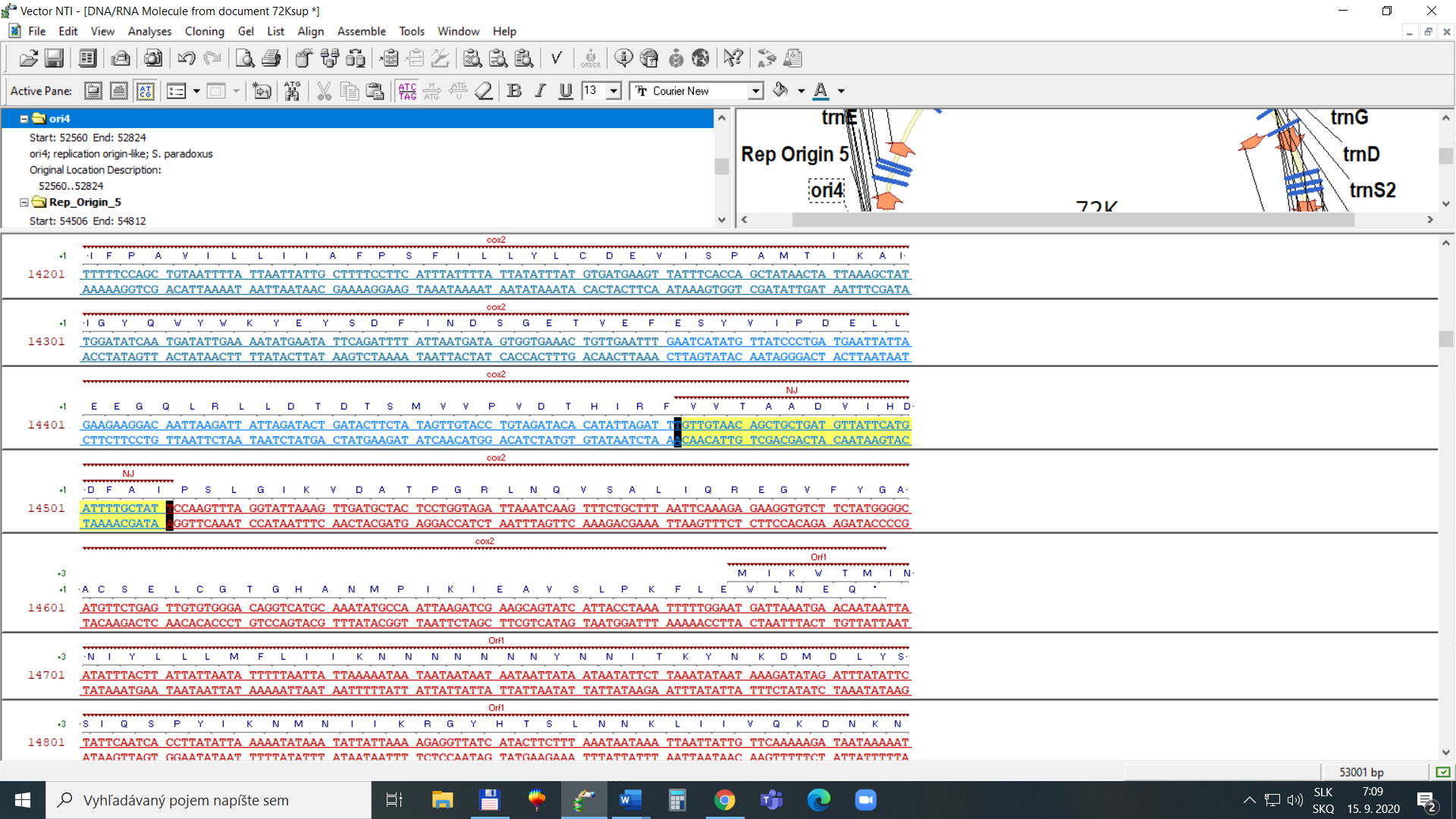


**5´junction**


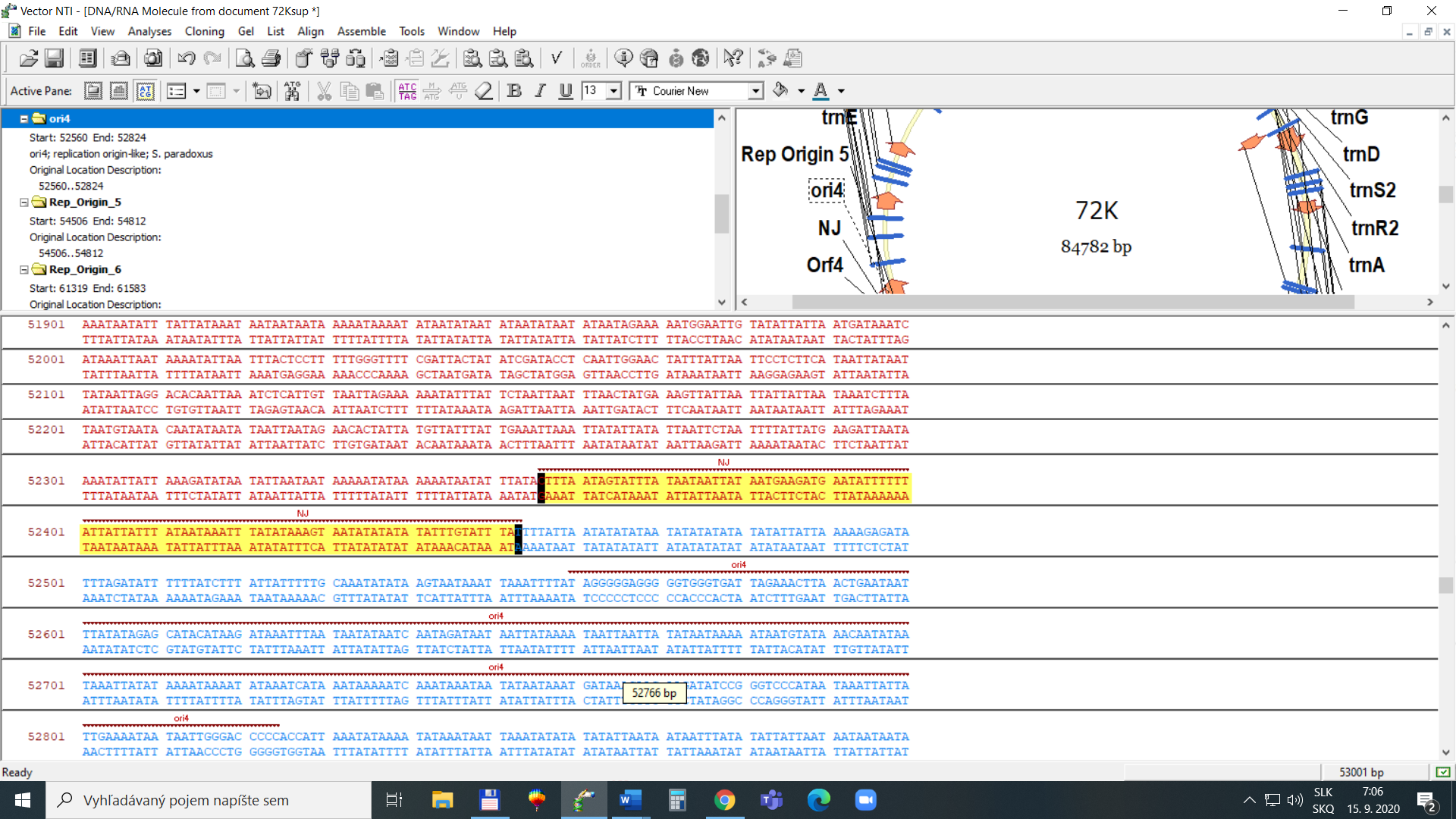


**3´junction**

**C. 4-R3 (72K) molecule**


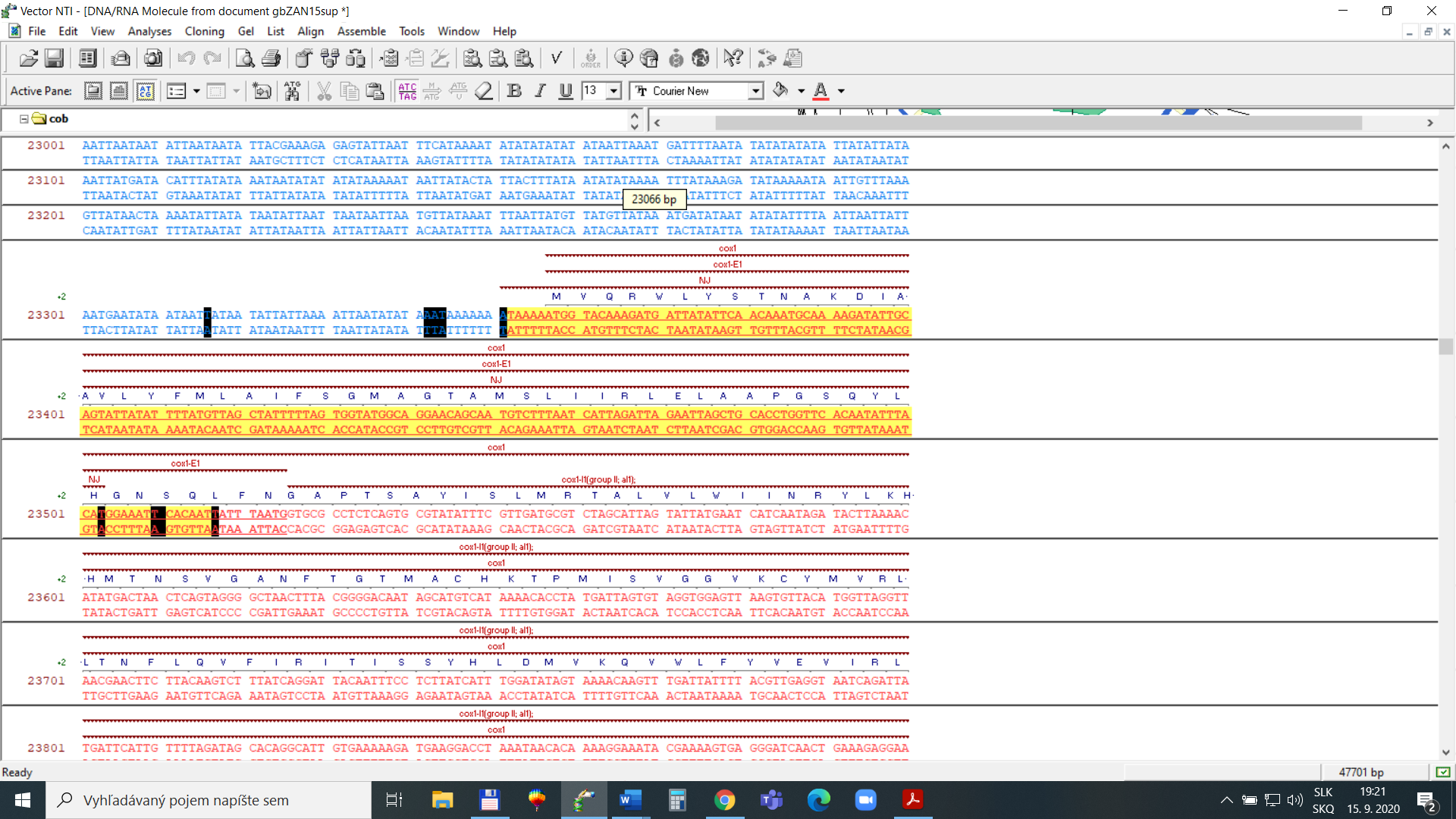


**5´junction**


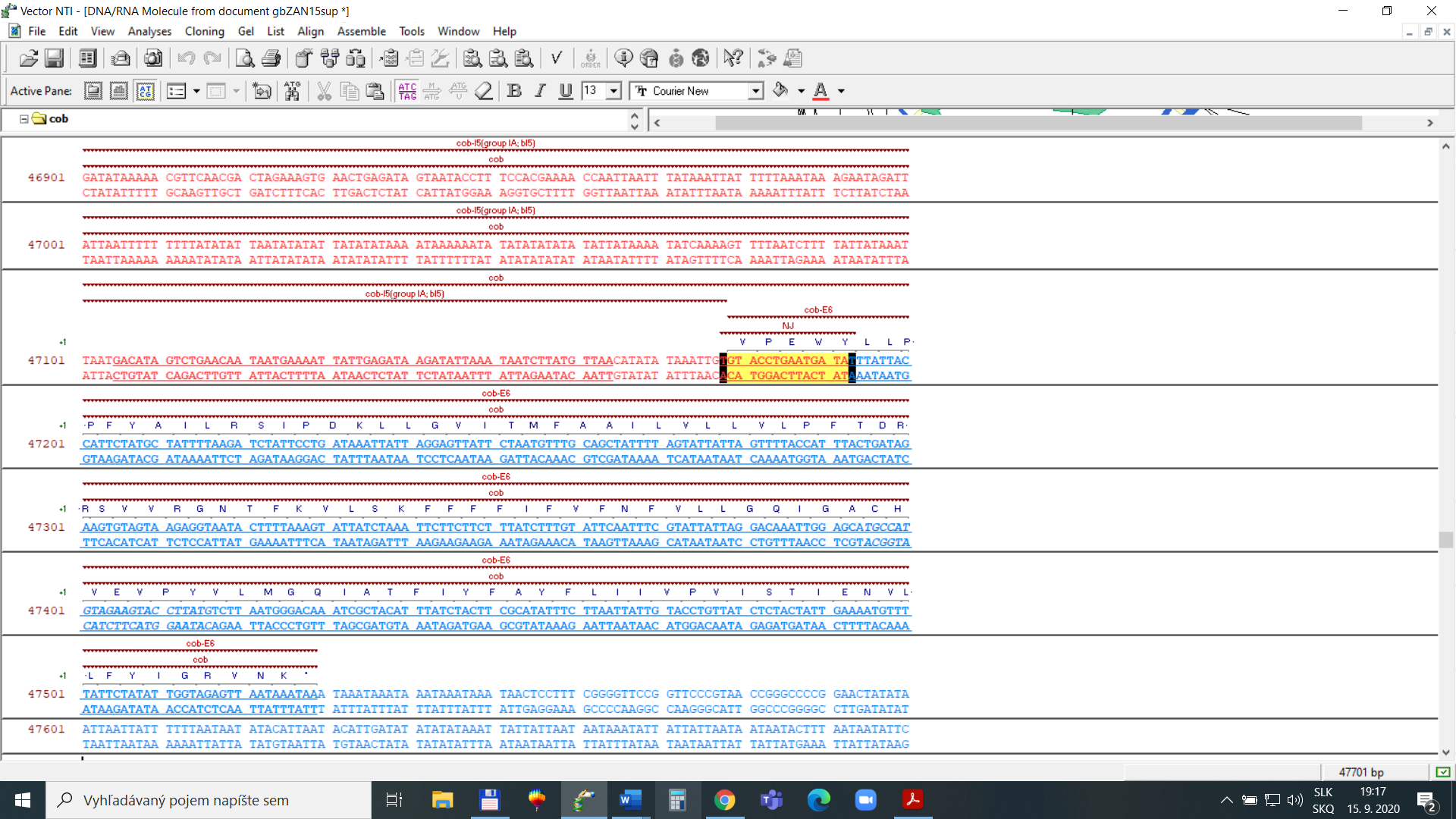


**3´junction**

**D. ZAN15 molecule**


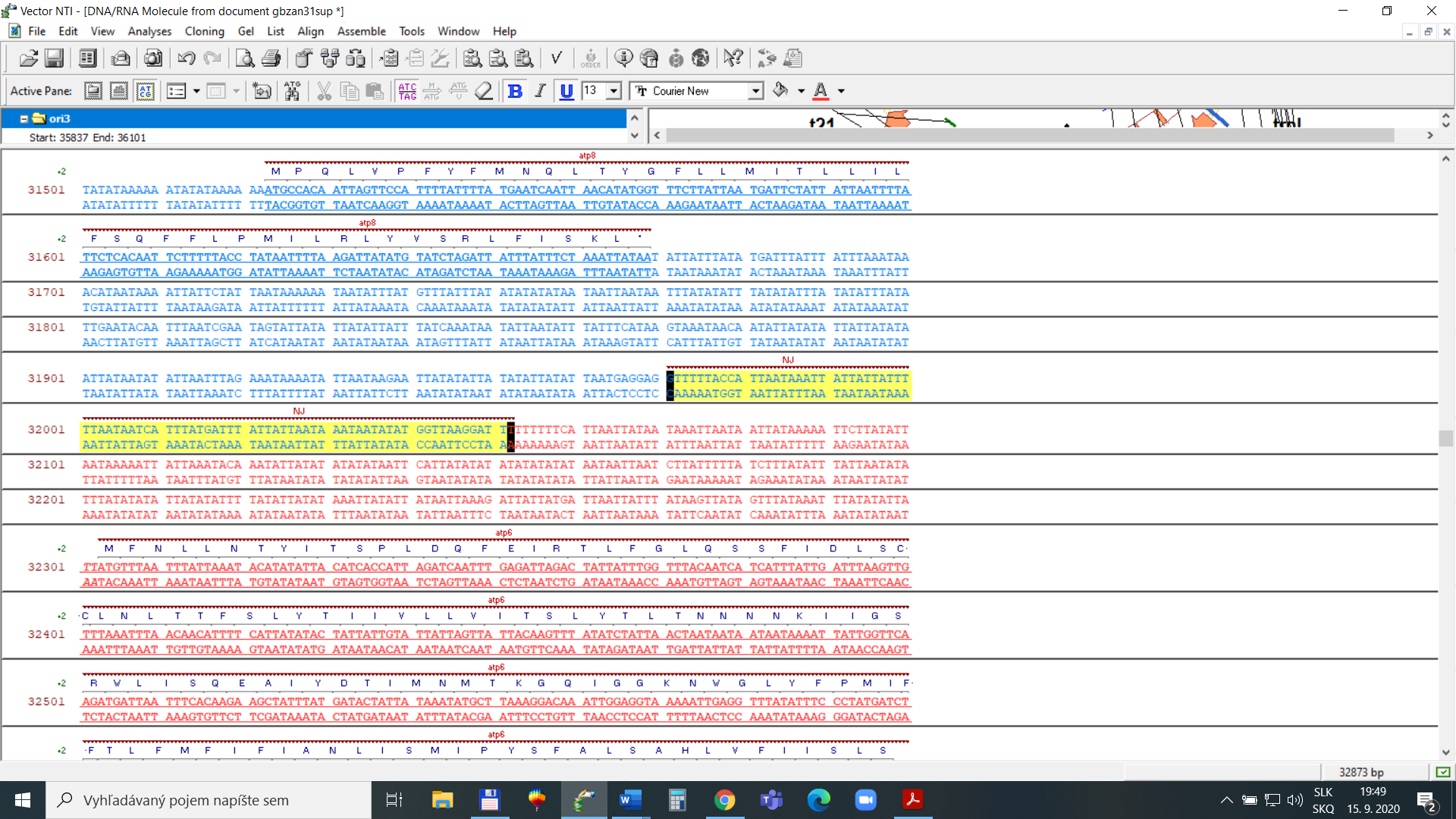


**5´junction**


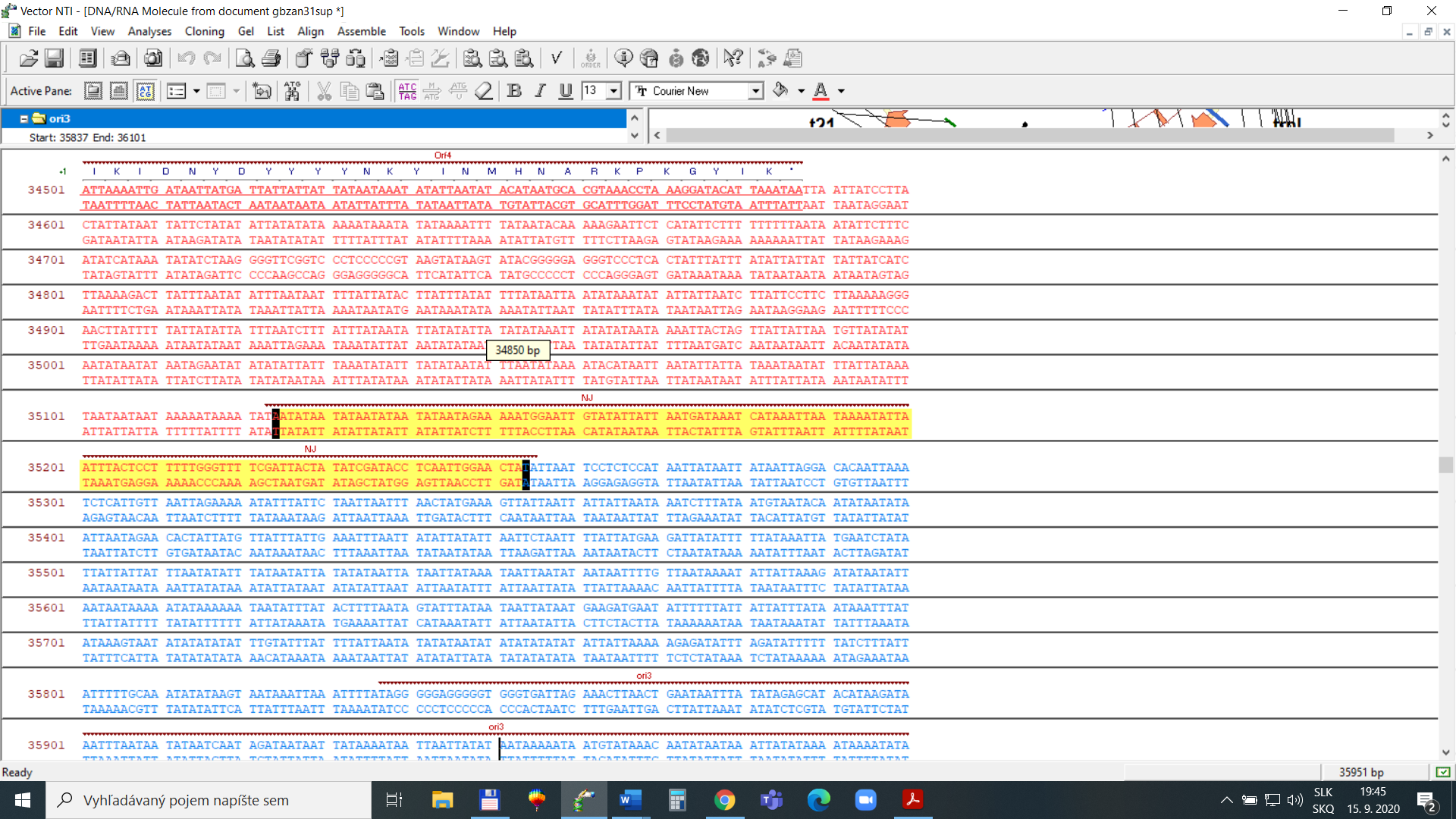


**3´junction**

**E. ZAN31 molecule**


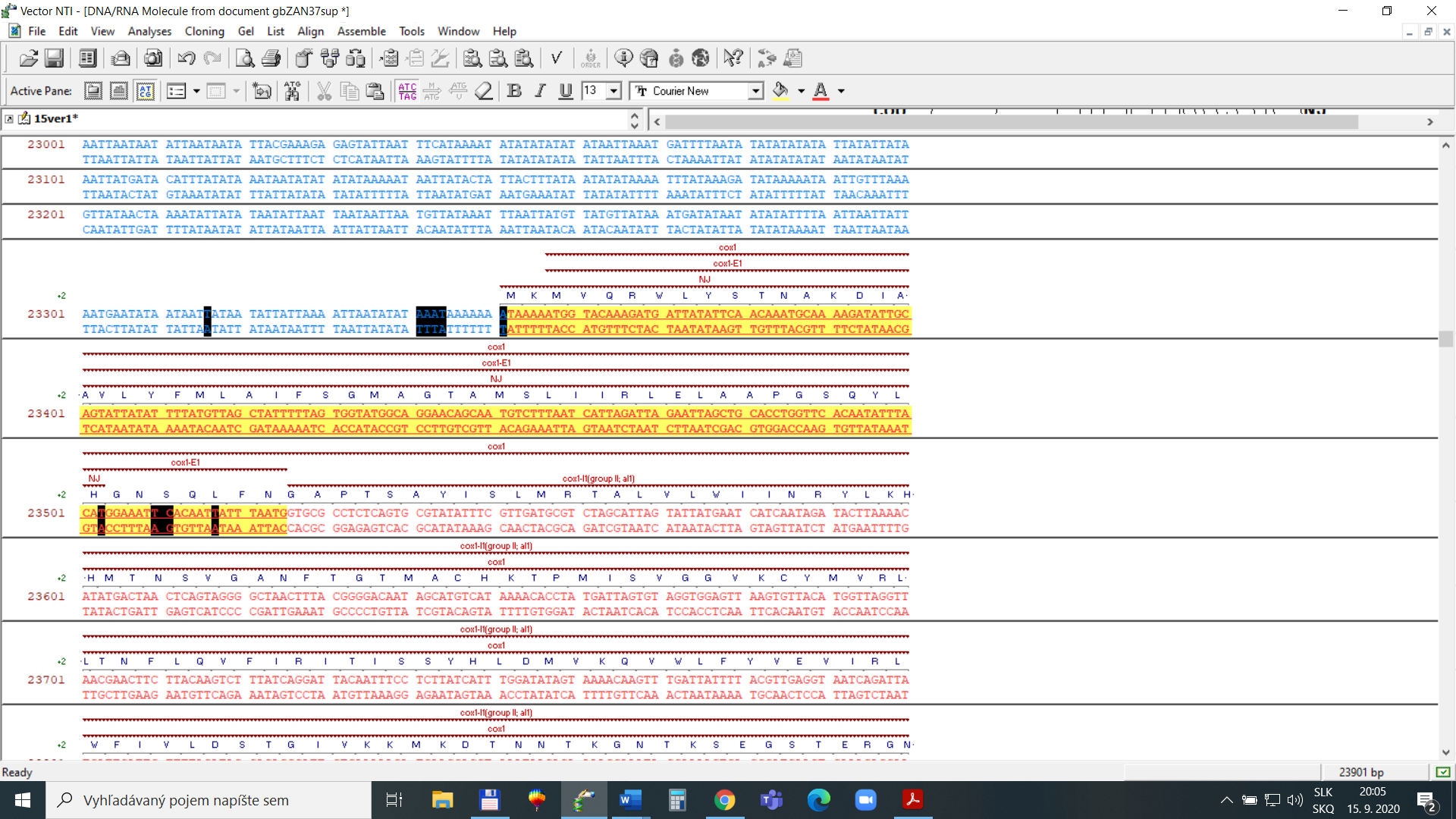


**5´junction**


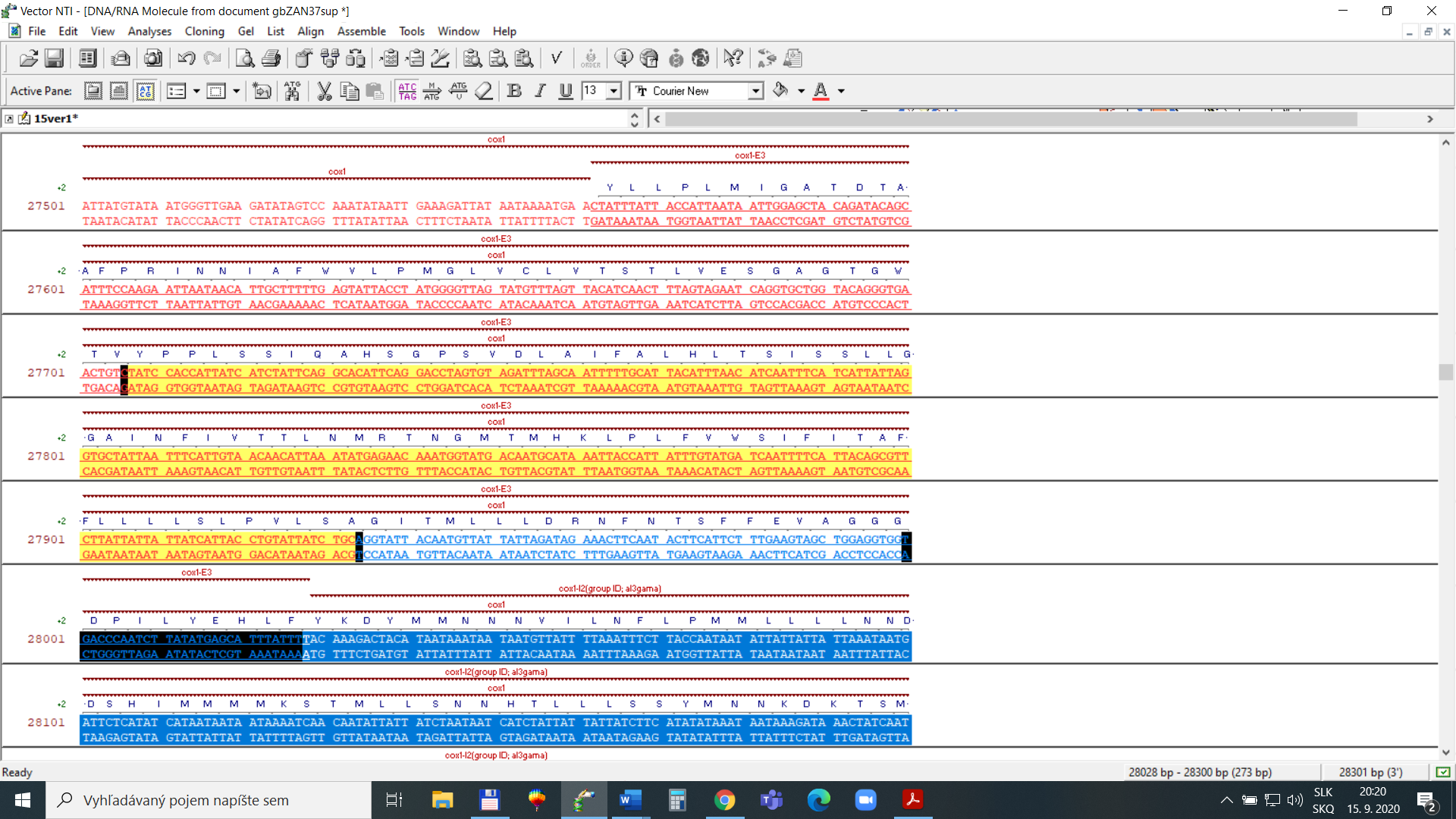


**3´junction**


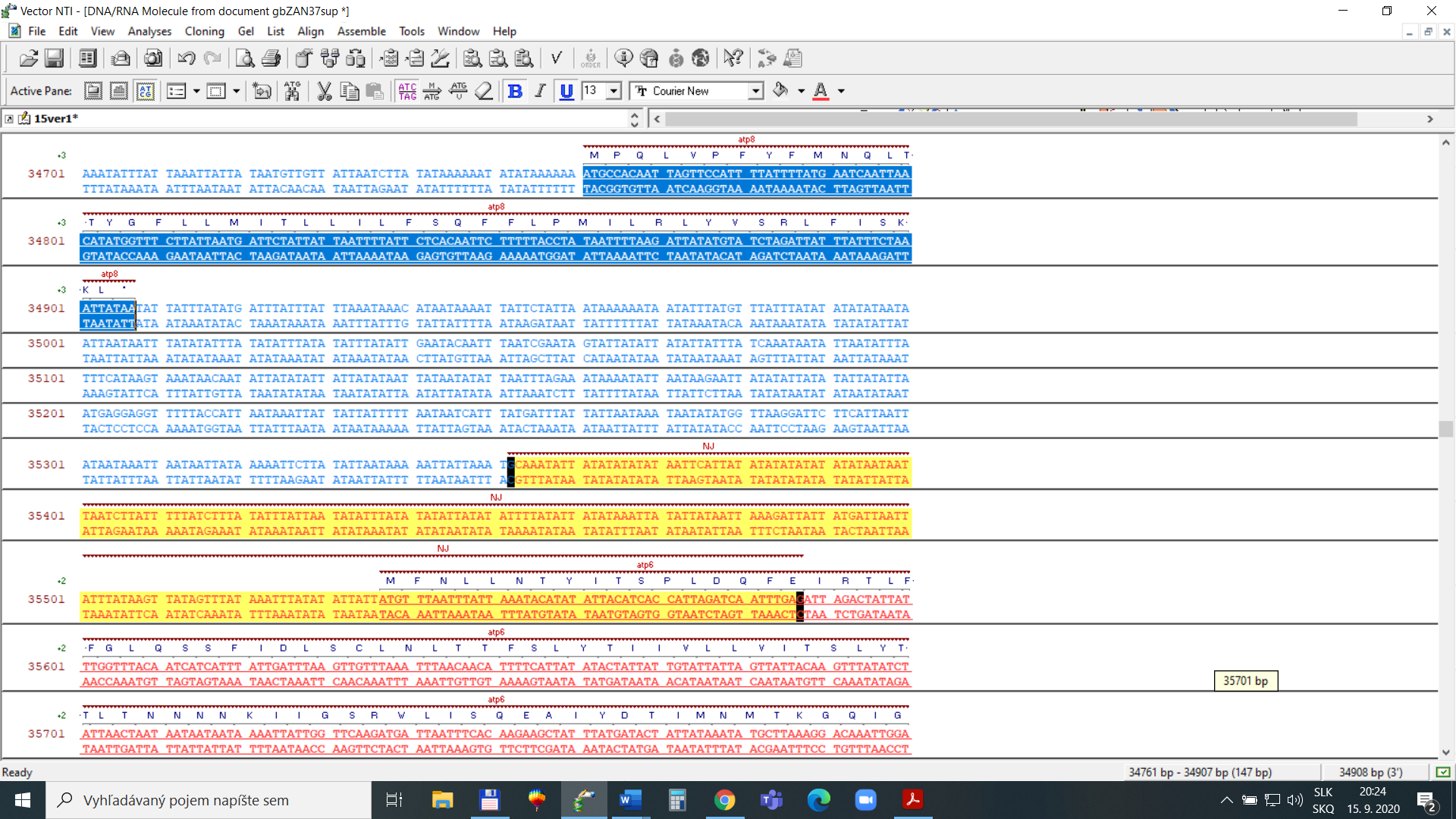


**5´junction**


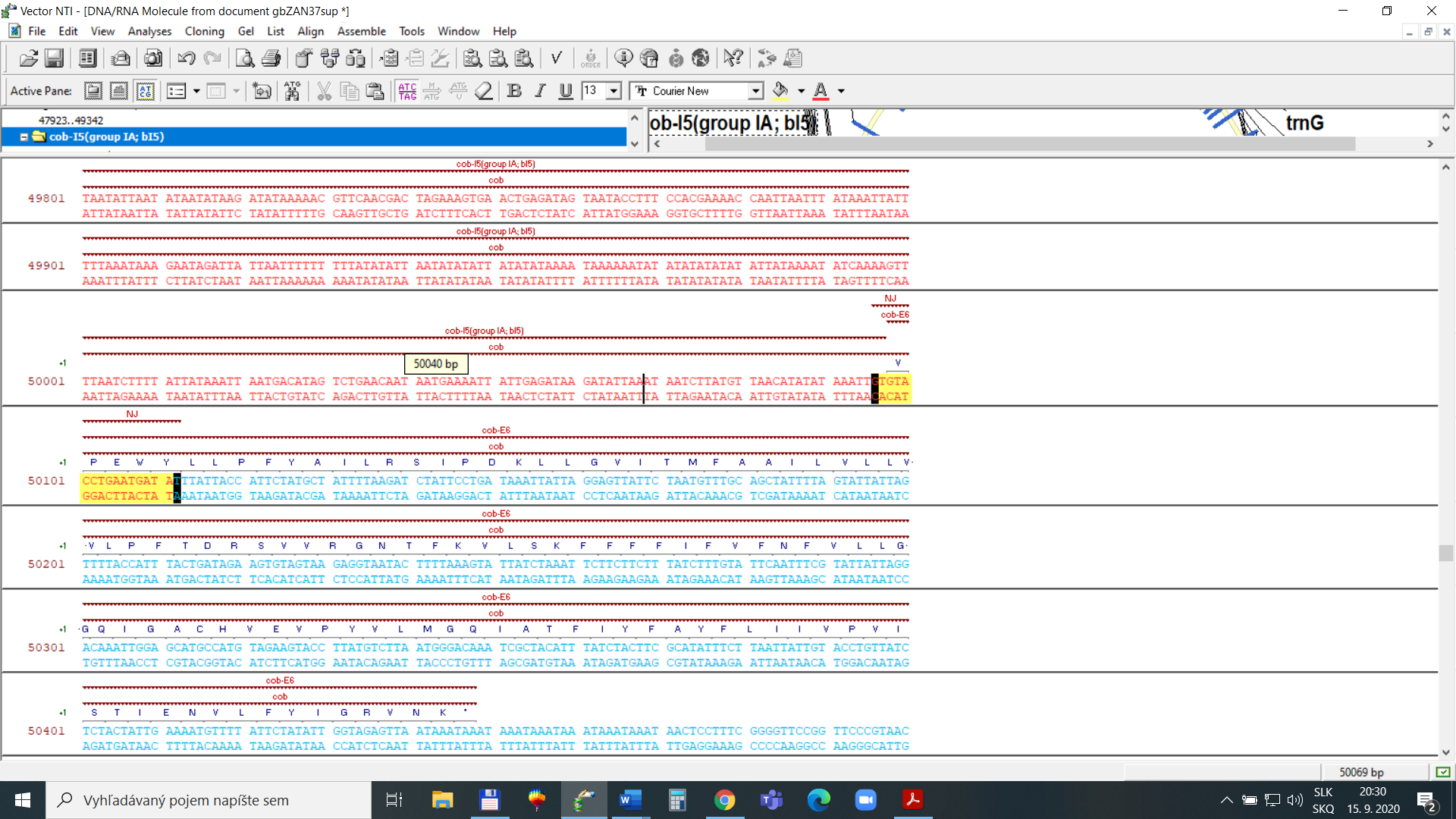


**3´junction**

**F. ZAN37 molecule**

**Fig. S6.** **DNA sequence of recombination sites.** **A. Hybrid 3-R1 molecule. B. Hybrid 6-R2 molecule. C. 4-R3 (72K) molecule. D. ZAN15 molecule. E. ZAN31 molecule. F. ZAN37 molecule.** *S. cerevisiae* DNA marked in red. *S. paradoxus* DNA marked in blue. NJ novel junctions in homologous region are highlighted in yellow. Species specific polymorphism is highlighted in black. Exons are bold and underlined.
